# Feeding Ecology of Northeast Atlantic Mackerel, Norwegian Spring-Spawning Herring and Blue Whiting in the Norwegian Sea

**DOI:** 10.1371/journal.pone.0149238

**Published:** 2016-02-19

**Authors:** Eneko Bachiller, Georg Skaret, Leif Nøttestad, Aril Slotte

**Affiliations:** Pelagic Fish Research Group, Institute of Marine Research (IMR), PO Box 1870, Nordnes, NO-5817, Bergen, Norway; Aristotle University of Thessaloniki, GREECE

## Abstract

The Norwegian spring-spawning (NSS) herring (*Clupea harengus*), blue whiting (*Micromesistius poutassou*) and Northeast Atlantic (NEA) mackerel (*Scomber scombrus*) are extremely abundant pelagic planktivores that feed in the Norwegian Sea (NS) during spring and summer. This study investigated the feeding ecology and diet composition of these commercially important fish stocks on the basis of biological data, including an extensive set of stomach samples in combination with hydrographical data, zooplankton samples and acoustic abundance data from 12 stock monitoring surveys carried out in 2005–2010. Mackerel were absent during the spring, but had generally high feeding overlap with herring in the summer, with a diet mainly based on calanoid copepods, especially *Calanus finmarchicus*, as well as a similar diet width. Stomach fullness in herring diminished from spring to summer and feeding incidence was lower than that of mackerel in summer. However, stomach fullness did not differ between the two species, indicating that herring maintain an equally efficient pattern of feeding as mackerel in summer, but on a diet that is less dominated by copepods and is more reliant on larger prey. Blue whiting tended to have a low dietary overlap with mackerel and herring, with larger prey such as euphausiids and amphipods dominating, and stomach fullness and feeding incidence increasing with length. For all the species, feeding incidence increased with decreasing temperature, and for mackerel so did stomach fullness, indicating that feeding activity is highest in areas associated with colder water masses. Significant annual effects on diet composition and feeding-related variables suggested that the three species are able to adapt to different food and environmental conditions. These annual effects are likely to have an important impact on the predation pressure on different plankton groups and the carrying capacity of individual systems, and emphasise the importance of regular monitoring of pelagic fish diets.

## Introduction

The Norwegian Sea (NS) is the feeding ground of some of the largest fish stocks in the world, including two of the ten with highest global yields [1], namely Norwegian spring spawning (NSS) herring (*Clupea harengus*) and blue whiting (*Micromesistius poutassou*). In addition, the abundant North East Atlantic (NEA) mackerel (*Scomber scombrus*) population spend the summer feeding in the NS. These planktivorous populations have substantial spatial and dietary overlap [2–4], and are often collectively referred to as the ‘pelagic complex’ in the NS. Fluctuations in abundance of these populations have been observed in the NS since the late 1960s [5–7], but since the late 1980s their combined abundance has increased steadily to form one of the highest biomasses on record [8,9], in particular due to an increase in the abundance of NEA mackerel [9–11]. The increased abundance means increased potential for interactions between the populations which in turn may have a strong ecological impact [12,13].

The plankton community of the NS is dominated by the calanoid copepod *Calanus* spp., amphipods and krill [8], all of which are preyed upon by the planktivorous fish [14]. The composition of the prey of the pelagic species in the NS has been investigated by several studies. Prokopchuk and Sentyabov [15] found *Calanus finmarchicus* to be the principal prey of mackerel in summer (June and July) 2001 and 2002, while *C*. *finmarchicus* was an important prey of herring only in July 2001 and June 2002, while in July 2002 appendicularians, amphipods and euphausiids dominated their diet. Such opportunistic predation on larger prey, complementing the usual calanoid-copepod-based diet, had already been observed by Dalpadado et al. [3]. On the other hand, appendicularians, amphipods and euphausiids were the main prey of blue whiting throughout the feeding season, so that there was a limited prey overlap with herring and mackerel [15]. The later study by Langøy et al. [16] confirmed this general picture of prey composition, and these authors also found that mackerel in particular were opportunistic, adjusting their feeding activity and diet to prey availability (see also [13]).

In spite of the potentially large overlap in diet composition, the species interactions are determined by the degree of spatial and temporal overlap between the populations. The potential spatial overlap between the populations is to some degree restricted by their different temperature and depth preferences. In summer mackerel prefer water temperatures above 8 °C [17], while herring and blue whiting are mainly found in water masses between 2 and 8 °C [18]. Meanwhile, blue whiting usually prefer deeper waters than the other two species [18]. In confirmation of this, a low horizontal overlap between herring and mackerel has been observed [16], and a modelling study by Utne et al. supported this finding, also finding a large horizontal overlap between herring and blue whiting [19]. While the traditional peak feeding season for herring and blue whiting is in May-June [3,20], for mackerel it is in July [17,21]. Utne et al. [18] observed that the three populations often utilized many of the same feeding areas in the NS between 1995 and 2006, but at different times, with high seasonal and inter-annual variability in the horizontal overlap. However, more recent studies have found rather strong overall spatial overlaps within the pelagic complex during the feeding season, probably due to variations in prey (e.g. *C*. *finmarchicus*) distribution [8,22–24] as well as growing population sizes and an earlier onset of the mackerel feeding migration [7,13,16,22].

Knowledge of the feeding ecology of the pelagic populations in the Norwegian Sea is essential for a proper understanding of important ecological functions like carrying capacity, distributional shift, competition and growth. However, information on the feeding ecology and diet composition of the major pelagic fish species in the NS is limited. No up-to-date studies of the area after 2006 exist, and little is known about spatial variations, potential effects of environmental conditions on feeding activity, or inter-specific interactions or annual and seasonal variations in the diet composition of these species.

This study investigated the feeding ecology and diet composition of the NEA mackerel, NSS herring and blue whiting during the spring and summer seasons from 2005 to 2010, these being the major feeding periods, and covering major areas where the three species potentially co-occur (Atlantic, Arctic and Coastal water masses). We also performed a detailed diet analysis for the area of the NS dominated by Atlantic water, which makes up the largest part of the feeding area and was therefore most extensively sampled. This is the first cross-season, multi-year study of the stomach fullness, feeding incidence, diet composition and diet overlap of the three most abundant and important ecological and commercial pelagic fish populations in the NS.

## Materials and Methods

In general, Institute of Marine Research (IMR), which is the responsible institution for monitoring Norwegian Spring Spawning herring, mackerel and blue whiting in Norway, and responsible for giving advice to managers in Norway, is given specific research quotas and special permission to sample these species at any location within the Norwegian economical zone by the Directorate of Fisheries, Bergen, Norway. Permission to sample the same species has been given by national authorities in Iceland and the Faroes for sampling within their economical zones. This is a general rule which applies to the annual monitoring of these populations.

Our study did not involve any endangered or protected species. No experimentation with animals was performed. No other ethical issues applied to the present research project. Special permissions or rules for sacrificing fish, from Institutional Animal Care and Use Committee (IACUC) or equivalent animal ethics committees, are at present non-existing in Norway for scientific fish sampling. Normally, the process of trawling and handling until biological sampling would lead to high mortality of the fish. However, if the fish were still alive after the trawling and handling process, they were quickly scarified by a hit to the head prior to biological analyses. Hence, fish were collected without unnecessary suffering, and their biological data was sampled and recorded according to standardized procedures described by Mjanger et al. [25].

The data were collected in the course of 12 surveys that covered large areas of the NS during May (spring) and July (summer) in 2005–2010 as part of an annual resource-monitoring programme, using both scientific and commercial vessels, depending on year and season (Table 1). All the data underlying the present study are available from the Dryad Digital Repository (doi:10.5061/dryad.f5r7f).

**Table 1:** Summary of sampling design and sample collection for different surveys.

Year	Season	Survey	Vessel	Period	N_CTD_	N_WP2_	Fish sampling			
							Fishing gear	Fishing depth	N_st (Dataset1)_	N_st (Dataset2)_
**2005**	**spring**	IESNS	R/V G.O. Sars	May 9—May 31	72	15 (0)	Pelagic trawl: Åkra trawl	0-450m	13	5
		IESNS	R/V Johan Hjort	May 1—May 31	123	-	Pelagic trawl: Åkra trawl	0-350m	3	
	** **						Pelagic trawl: Harstad trawl (29x29 m)	0-350m	2	
	**summer **	NESSNS	F/V Libas	July 18—July 29	-	-	Pelagic trawl: blue whiting trawl	0-40m	31	15
		NESSNS	R/V G.O. Sars	July 1—July 29	44	-				
**2006**	**spring**	IESNS	R/V G.O. Sars	May 1—May 30	69	36 (36)	Pelagic trawl: Åkra trawl	0-450m	43	24
							Pelagic trawl: Harstad trawl (29x29 m)	0-400m	2	
	** **	IESNS	R/V Johan Hjort	May 1—May 31	103	-			-	-
	**summer **	NESSNS	F/V Endre Dyroey	July 15—Aug. 3	62	17 (0)	Pelagic trawl: blue whiting trawl	0-40m	23	12
**2007**	**spring**	IESNS	R/V G.O. Sars	May 1—May 31	59	27 (27)	Shrimp trawl	0-450m	3	
							Pelagic trawl: Harstad trawl (29x29 m)	0-450m	5	1
	** **						Pelagic trawl: Åkra trawl	0-550m	65	31
	**summer**	IESSNS	F/V Eros	July 1—July 29	60	-			-	-
		IESSNS	F/V Libas	July 16—Aug. 3	58	35 (0)	Pelagic trawl: blue whiting trawl	0-300m	41	18
**2008**	**spring**	IESNS	R/V G.O. Sars	May 12—May 29	72	-			-	-
		IESNS	R/V Johan Hjort	May 4—May 31	69	-			-	-
	** **	IESNS	F/V Nybo	May 6—May 17	-	15 (0)	Pelagic trawl: Greater Argentine trawl (608 m)	0-450m	18	7
	** summer**	SALSEA	F/V Eros	July 28—Aug. 7	31	-	Pelagic trawl: salmon trawl (60x10 m)	0-20m	8	5
**2009**	**spring**	IESNS	R/V G.O. Sars	May 31	11	-			-	-
	** **	IESNS	R/V Johan Hjort	May 1—May 31	66	9 (9)	Pelagic trawl: Åkra trawl	0-500m	10	5
	**summer**	SALSEA	F/V Libas	July 18—July 29	44	2 (2)	Pelagic trawl: salmon trawl (60x10 m)	0-20m	3	1
	** **	SALSEA	F/V Eros	July 19—Aug. 4	47	15 (15)	Pelagic trawl: salmon trawl (60x10 m)	0-20m	17	7
**2010**	**spring**	IESNS	R/V G.O. Sars	May 7—May 29	64	3 (0)	Pelagic trawl: Åkra trawl	0-350m	3	2
	** **	IESNS	R/V Johan Hjort	May 1—May 31	141	-			-	-
	** summer**	IESSNS	F/V Libas	July 15—Aug. 18	92	62 (62)	Pelagic trawl: blue whiting trawl	0-300m	67	32

N_CTD_ and N_WP2_ denote the number of hydrographic and plankton sampling stations respectively; in N_WP2_ values in brackets indicate the number of stations in which, in addition to the total plankton biomass, size-fractioned (i.e. dry weight of <1000 μm, 1000–2000 μm and >2000 μm zooplankton) measurements were obtained. N_st (Dataset1)_ is the number of stations with available stomach content information in all water masses (including stations where single species were caught); N_st (Dataset2)_ is the number of stations within the Atlantic water mass and with spatial overlap between at least two of the target predator species. IESNS: International Ecosystem Survey in the Norwegian Sea; NESSNS: National Ecosystem Summer Survey in the Norwegian Sea; IESSNS: International Ecosystem Summer Survey in the Nordic Seas; SALSEA: Advancing understanding of Atlantic Salmon at Sea (EU project).

**Fig 1:**
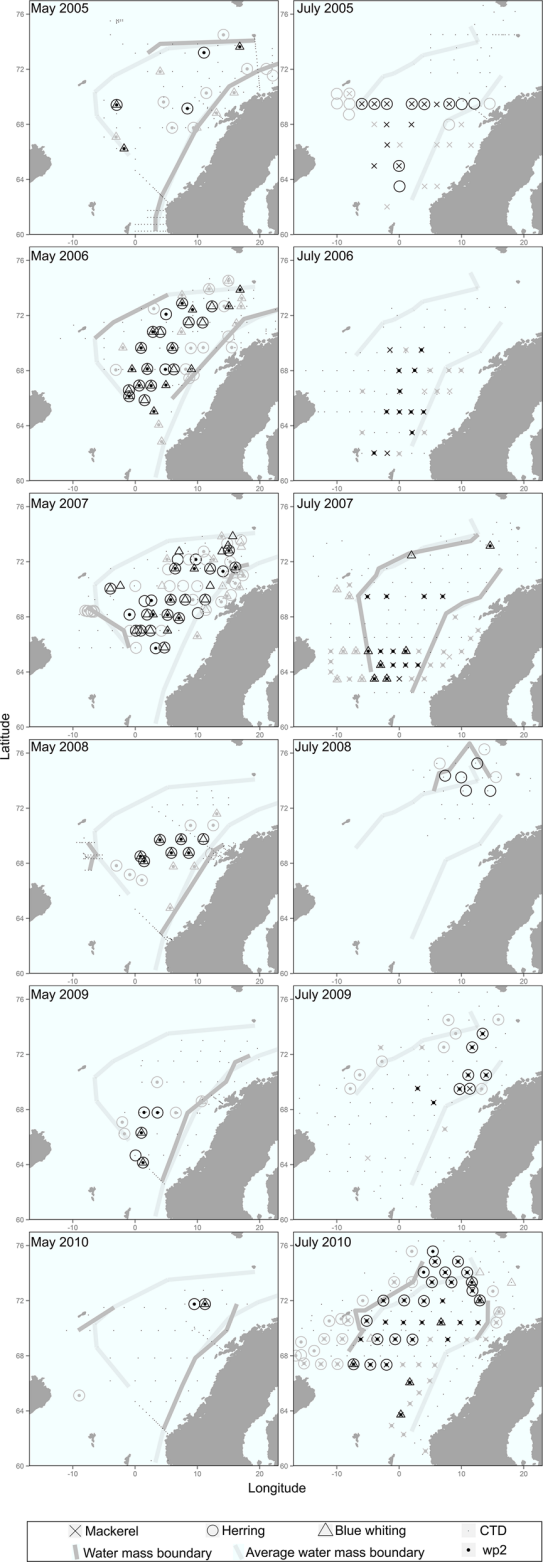
Fish sample distribution in May and July for 2005–2010. Small and large dots indicate CTD and WP2 (plankton) sampling stations, respectively. Black symbols represent stations included in both the general diet overview analysis (‘Dataset1’, Table 1) and the annual and inter-specific diet comparisons (‘Dataset2’, Table 1), while grey symbols are those excluded from the second analysis. Stations marked in black but showing single species indicate the presence of at least two predator species on the haul, the stomach content of only one of which was sampled. Dark grey lines indicate water mass boundaries for each year and season, and light grey lines the average boundaries for each season.

Fish and zooplankton samples were obtained at stations along predefined survey transects, covering eastern, central and southern parts of the NS (Fig 1). In addition, Conductivity, Temperature and Depth (CTD) measurements were obtained from research vessels. The spatial coverage of the survey differed from year to year, as did the sampling effort, and this needed to be taken into consideration in the analyses described in the following sections. Samples were obtained from 1287 CTD stations, 236 zooplankton sampling stations and 357 fishing stations (Table 1).

### Environmental variables and water mass definition

CTD casts were carried out using Seabird 911 and SAIV SD 204 instruments from the surface down to 500 m (Table 1). Following the methodology of Broms et al. [24], salinity at 20 m and sample location relative to the Atlantic water mass were used to associate each sampling station with one of three different water mass types: Coastal water mass (salinity <35 and sampling location east of the Atlantic water mass), Atlantic water mass (salinity ≥35) and Arctic water mass (salinity <35 and sampling location west of the Atlantic water mass). Water mass boundaries were defined for each year and season. Average boundaries based on all sampling years together were used in cases where the CTD stations did not cover the entire sampling area (Fig 1).

### Zooplankton data

Zooplankton samples were collected at some of the fishing stations (Table 1; Fig 1) using a WP2 plankton net with a diameter of 56 cm and mesh size of 180 μm. The net was hauled vertically from a depth of 200 m to the surface, following the standard procedures of the Institute of Marine Research (IMR, Norway [14]). The plankton hauls were carried out immediately before the start of a trawl haul. At most stations, each zooplankton sample was split into three size fractions using 180μm, 1000μm and 2000μm sieves. Organisms from the largest size fraction (i.e. >2000μm) were counted, separated and identified to the lowest possible taxonomic group. Each plankton fraction (and group separated from the large fraction) was then oven-dried at 70°C for more than 24 h to constant dry weight and weighed on a micro-balance to the nearest 1 mg. At some stations, zooplankton samples were collected, dried and weighed following the same procedure but without size fractionation. Either the sum of the dry weights of all the fractions or the total dry weight of the sample was used to estimate zooplankton biomass at each station. From the May surveys, biomass measurements of total zooplankton were obtained from 105 stations (2005–2010), from which biomass measurements of fractioned zooplankton were made in 72 stations (2006–2007, 2009). From the July surveys, total zooplankton biomass was obtained for 131 stations (2006–2007, 2009–2010), with additional size-range information obtained for 77 of these (2009–2010) (Table 1).

In order to take into account the effect of the number of stations on comparisons of the zooplankton biomass across different seasons and water masses, the average biomass (dry weight) of each size fraction of zooplankton (*fractionDW*) was weighted by the total zooplankton biomass for a given water mass and season, according to the following equation:

$$fractionDW=\frac{\sum_{st=1}^{n}fractionDW_{st}sumDW_{st}}{\sum_{st=1}^{n}sumDW_{st}}$$ (1)

where *fractionDW*_*st*_ is the zooplankton dry weight (g m^-2^) for the given fraction at station *st*, and *sumDW*_*st*_ is the total dry weight of zooplankton (i.e. all fractions) at station *st*.

### Seasonal variations in zooplankton biomass distribution

Seasonal variations were assessed separately for each zooplankton size fraction as well as for the total zooplankton biomass. Preliminary data exploration recommended in Zuur et al. [26] determined a high collinearity between ‘year’, ‘season’ and ‘water mass’, as well as a lack of enough size-fractionated zooplankton samples from coastal and arctic water masses (see Table 1) to use multivariate methods. Accordingly, mean values of zooplankton population biomass (each zooplankton size fraction separately as well as the whole size range) were compared within the Atlantic water mass between seasons, after pooling all the years together (F tests).

### Fish abundance estimates

Fish abundance estimates were based on acoustics or trawl hauls and were averaged and projected into 1° latitude by 1° longitude grids. For herring and blue whiting, acoustic estimates in tonnes per grid were based on backscatter from calibrated 38 kHz echo-sounders. The data were resolved at 1 nm and the backscatter identified and allocated to the given species on the basis of expert evaluation, and established target strength conversions from echo backscatter to fish biomass were used (see [9] for further details). For mackerel, estimates were based on catch-per-unit-effort (CPUE).

### Temperature conditions for mackerel, herring and blue whiting

In order to investigate the effect of changes in temperature, the ambient temperature at each fish-sampling station was considered, according to the following equation:

$$ambientT=\frac{\sum_{st=1}^{n}T_{D}B_{p}}{\sum_{st=1}^{n}B_{p}}$$ (2)

where *T*_*D*_ is the temperature at the fishing depth (defined based on acoustic observations prior to the sampling) of station *st* and *B*_*p*_ is the total predator biomass in the corresponding (1°latitude x 1°longitude) grid. These values, weighted to the fish abundance in the individual grid cell [18], determined the ambient temperature (*ambientT*) of the species averaged over water mass, season and year.

### Fish sampling and diet analysis

#### Biological sampling

NEA mackerel, NSS herring and blue whiting were captured in May (spring) and July (summer) by pelagic trawl, using different gears depending on the survey and vessel (Table 1; Fig 1). In May, trawling for herring and blue whiting was performed following acoustic registrations from 15 to 300 m depth, while in July, almost all trawling was done at depths of 5 to 50 m, where a large majority of mackerel and herring feed during the summer. At each station, 10 randomly selected individuals per species were sampled from the catch whenever the catch size permitted.

Fish were sized and weighed so that Fulton’s condition factor (CF) could be determined, according to *CF = (W/L*^*3*^*)100*, where *W* is wet weight (g) and *L* is total length (cm). Stomachs were extracted from the fish and preserved frozen.

#### Stomach content analysis

In the laboratory, a stereomicroscope was used for the identification of stomach contents. Only material contained in the stomachs was considered, with the contents of the intestine and esophagus being discarded in order to reduce potential bias caused by different rates of ingestion and gut passage times or cod-end feeding [27]. During processing, stomach contents were carefully taken apart and all identifiable prey counted and specified to the lowest possible taxonomic group, not including broken parts of appendixes in the counting, and categorized into 41 groups. For the graphical presentations prey groups were merged into the following 13 groups: *C*. *finmarchicus*, other calanoids, copepod remains, Euphausiacea ord., Decapoda ord., *Themisto* spp., other amphipods, crustacean remains, Gastropoda cl., Appendicularia cl., Actinopterygii cl., other remains and unidentified remains.

Prey species and groups from each stomach were oven-dried separately at 70°C for more than 24 h to constant dry weight and weighed by micro-balance to the nearest 1 mg.

Feeding incidence (FI) was calculated as the ratio in percentages between the number of sampled fish with any stomach content and the total number of sampled fish.

Feeding intensity was assessed using stomach fullness degree (SFD) calculations as a proxy. The SFD was defined as the sum of the weights of all the prey in a stomach (mg) divided by the total length of fish (mm). In that respect, a preliminary analysis did not show any trend in the SFD with the total weight of each fish, neither on the SFD calculated by dividing the weight of stomach contents by the total weight of each fish, with the total length of fish (which, at the same time, was closely related to the weight, due to allometry). This approach is therefore believed to be a useful estimator of feeding intensity [28], as it excludes the effect of fish size and avoids subjectivity problems expected from visual stomach fullness scaling methods such as using the trophometer [29] or the 1–5 scale of stomach fullness (IMR, [16]).

#### Data analysis (I): general overview of feeding related variables and diet composition

The prey composition in the diet of different predator species was represented as percentages of the total weight in stomach contents. In order to take into account the effect of predator abundance on prey ingestion, mean prey weight (*preyDW*) was weighted according to estimated predator abundance in a given area, season and year, according to the following equation:

$$preyDW=\frac{\sum_{st=1}^{n}preyDW_{st}predABD_{st}}{\sum_{st=1}^{n}predABD_{st}}$$ (3)

where *preyDW*_*st*_ is the mean prey dry weight (mg fish^-1^) in stomach contents at station *st*, and *predABD*_*st*_ is the estimated abundance of the predator species in the quadrant corresponding to station *st* (see section 2.3).

In order to obtain a general overview of the diet, the total zooplankton availability (by size ranges) and the diet composition of small pelagic species were described for the different water masses and seasons, considering all the years together.

Thereafter, in order to determine the influence of fish length and environmental conditions on the feeding and condition factor of the three species, different statistical models were fitted and evaluated. As in case of zooplankton seasonal variation analyses, the process of data exploration, model selection and model validation was made according to Zuur et al. [26]. ‘Temperature’ (at the maximum fishing depth), ‘fish length’ and ‘fish abundance’ were included as covariates for explaining variations in CF, SFD and FI. In addition, ‘season’ (in cases where data were available for both May and July) was added as an explanatory factor, and ‘year’ as either a fixed or random effect. The variables were averaged for each station and a model was fitted separately for each species and response variable. In a first approach, generalized linear mixed modelling (GLMM) was applied with ‘year’ added as a random effect. If model validation showed non-random patterns in the residuals over the years, ‘year’ was added as an additional fixed effect in a generalized linear model (GLM) approach. In the cases of CF and SFD, a Gaussian distribution of the error terms was assumed. In the case of FI, a binomial distribution of the error term was assumed. In a preliminary analysis, ‘zooplankton abundance’ was also included as explanatory variable for SFD and FI, which then reduced the number of stations available for the analysis to about one third (108 vs. 350 stations, Table 1). Zooplankton abundance did not have any significant effect on either SFD or FI, and the results are not presented here.

#### Data analysis (II): inter-specific comparisons and annual variations in diet composition

Diet width was estimated in order to determine how many prey groups each species exploited, and whether any of the species displayed more generalist or more opportunistic feeding behaviour. As defined in Langøy et al. [16], the diet width was estimated as the average number of zooplankton species or groups (based on detailed diet characterization, i.e. considering 41 prey groups defined in a previous section) that made up more than 10% of the weight of the diet of each fish species per station. Results were then used to calculate the average diet width per species, season and year.

In order to investigate possible inter-specific differences in SFD, FI and diet width, a two-way ANOVA was used for each response variable and each season separately, considering ‘species’ and ‘year’ (including the interaction between them) as explanatory variables.

In order to assess the inter-specific and annual differences in diet composition, and reduce the error of spatial heterogeneity in the analysis (i.e. the number of samples from Arctic and Coastal water masses was low and variable, Fig 1), a selection of stations at which at least two of the three predator species had been caught was made. The diet composition was thus investigated in different seasons and years, considering the stations within the Atlantic water mass showing co-occurrence between the species. This selection comprised 165 stations (635 mackerel, 827 herring and 621 blue whiting), which comprised 46% of the stations included in the first general diet composition analysis (52% of mackerel, 49% of herring and 70% of blue whiting fish samples) (‘Dataset 2’; Table 1, Fig 1).

On the other hand, the overlap in resource use between the pelagic planktivorous species was assessed in the same selection of stations, using Pianka’s [30] index of niche overlap:

O=∑pi,jpi,k[(∑pi,j2)(∑pi,k2)]12 (4)

where *O* is the overlap index between the two species *j* and *k* expressed as a value between 0 and 1, where 0 means no overlap and 1 complete overlap in diets. *p*_*i*,*j*_ and *p*_*i*,*k*_ are the proportions of the *i*^th^ prey group in the diets of species *j* and *k*, respectively. For diet overlap comparisons the stomach contents were categorised into 5 prey groups: Copepoda subcl. (all copepods grouped), Euphausiacea ord., Amphipoda ord., Appendicularia cl. and others. To test for significance, the proportion of a given prey group in a given diet was randomized according to the Randomization Algorithm (RA2) defined by Lawlor [31] and iterated 1000 times for each comparison of diet overlap. Lawlor [31] described four randomization algorithms (RA1-RA4) for niche overlap, in which the zero states (the empty prey groups) and the niche breadth (the degree of utilization of a prey group) can be either relaxed or retained. Under RA2, the zero states are retained (i.e. empty prey groups from the stomach samples remain empty in the simulations), while niche breadth is relaxed (i.e. the proportion in the diet of each non-empty prey group is replaced by a uniform value between 0 and 1). As in the case of Langøy et al. [16], RA2 was considered to provide the most realistic reflection of the Norwegian Sea pelagic system because some of the prey groups would be unavailable to fish in certain areas, due to the patchy distribution of the plankton [14], while none of the fish species were assumed to have constraints on the utilization of the prey groups that were actually present.

Finally, in order to investigate whether co-occurrence influenced prey selection and diet overlap, paired-comparisons of diet overlap were made, considering stations within the Atlantic water mass with and without co-occurrence between the compared species, respectively. This analysis was performed on all the years taken together, but separated by seasons.

The software packages *R v*. *3*.*0*.*2* [32] and *ggplot2 v*. *1*.*0*.*0* [33] were used for all analyses and graphical representations, respectively, except for the diet overlap. The functions and packages used for the statistical models were the following: glm function of the stats package for GLMs, lme function of the nlme package for GLMM with Gaussian distribution of the error term, and the glmmPQL function from the MASS package for GLMM with binomial error distribution. A forward model selection approach based on the AIC criterion was used in order to choose the optimal model. Diet overlap analysis was made using *EcoSim Professional v*.*1*.*2d* [34].

## Results

### Zooplankton distribution (prey availability)

**Fig 2:**
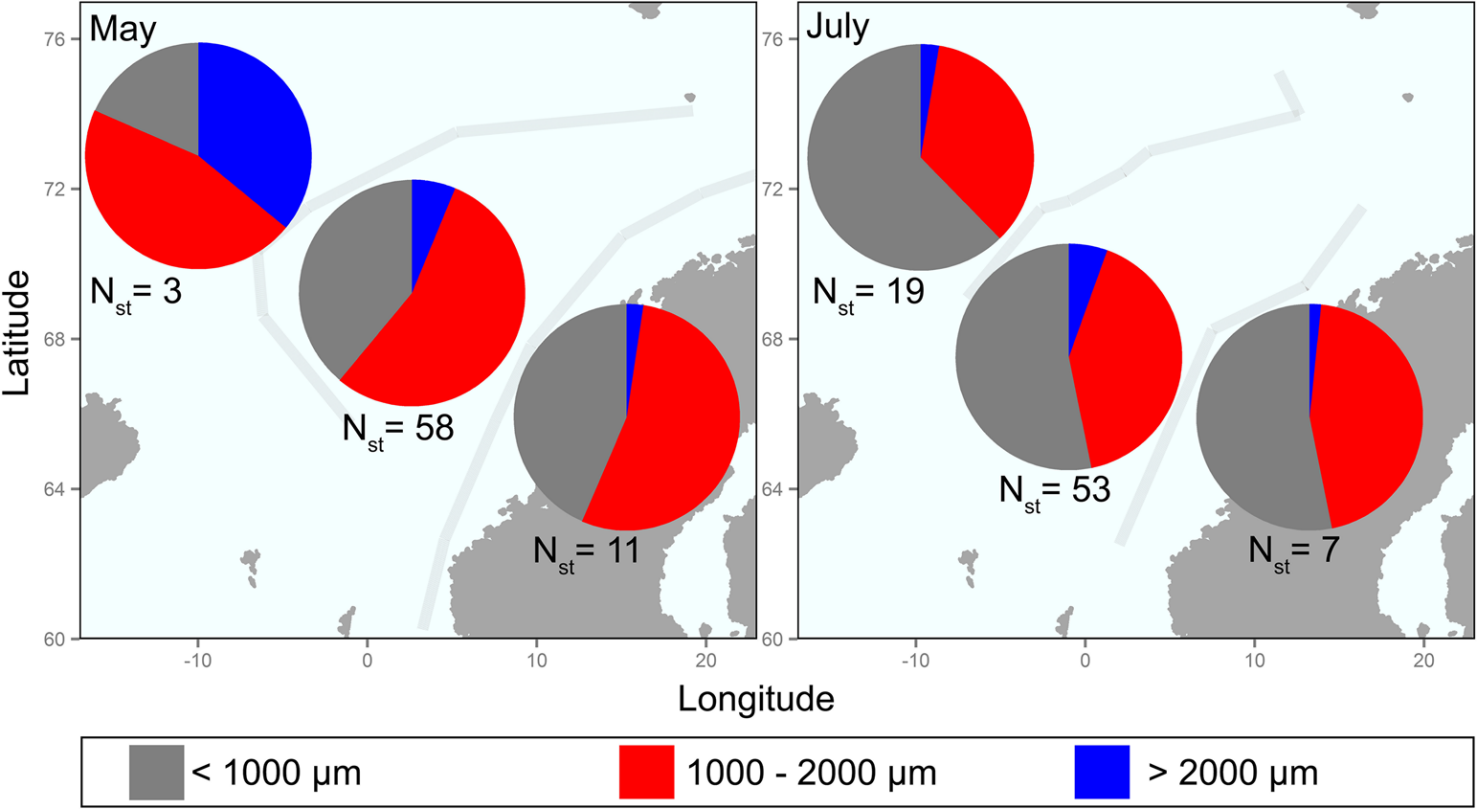
Average zooplankton biomass distribution (dry weight, g m^-2^) ranged by size (<1000 μm; 1000–2000 μm; >2000 μm) in May and July. All sampling years were analysed together (i.e. ‘Dataset1’ in Table 1; all stations in Fig 1). Light grey lines represent the average boundaries for each season. N_st_ indicates the number of stations in each water mass.

Zooplankton in the size range <2000 μm comprised the highest percentage of the total biomass both in May and July for all water masses (Fig 2). In May, 1000–2000 μm prey were the most abundant, while in July the smallest organisms (<1000 μm) were most abundant. The largest zooplankton (>2000 μm) contributed generally little to the total biomass, with particularly low contributions in coastal water mass. An exception seemed to be the Arctic water mass in May, where a low percentage of the smallest fraction and a higher percentage of the largest zooplankton was found (Fig 2). Distribution of zooplankton is presented as supplementary material (S1 Fig).

### Seasonal variations

Within the Atlantic water mass, and considering data from all the available years together (see section 2.2), the smallest fraction showed significantly higher biomass in summer (<1000 μm zooplankton: *F* = 36.53; *d*.*f*. = 1; p < 0.001). In contrast, a depletion of zooplankton might be suggested for larger zooplankton from spring to summer, although the difference was marginally not significant for 1000–2000 μm zooplankton (*F* = 2.02; *d*.*f*. = 1; p = 0.06) and clearly not significant for > 2000 μm zooplankton (*F* = 0.39; *d*.*f*. = 1; p = 0.53). Considering the whole size range no significant difference was observed between seasons (*F* = 3.06; *d*.*f*. = 1; p = 0.62).

### Fish distribution

Mackerel were only caught during the July surveys (summer) and in high abundances especially within the Atlantic water mass. Catches with particularly high quantities of mackerel were patchily distributed (S2A Fig). Mackerel were caught in waters with similar ambient temperatures as herring: between 7 and 13 degrees. It should be noted that mackerel were caught at several stations in Arctic waters in 2010 where the mean ambient temperatures were as low as 2.42 degrees (Table 2A). Herring distribution as recorded with acoustics was generally wide within the Atlantic water mass throughout the sampling years, even more in May (typically in waters between 3 and 7 degrees, Table 2A) than in July. The locations with the highest amounts of herring recorded were patchily distributed (S2B Fig). Blue whiting were generally distributed within the Atlantic and coastal water masses in both May and July (S2C Fig) and were distributed within a narrower ambient temperature range than the other species, occurring in waters from 4 to 7°C in almost all seasons, years and water masses (Table 2A).

**Table 2:** **(A) Number of samples (N), total length of fish (cm), and ambient temperature at the maximum fishing depth (°C), and (B) Fulton’s condition factor (CF), stomach fullness degree (SFD) and feeding incidence (in percentages), for May and July, in different years**.

**A**																
**Year**		**N**_**fish**_ **(N**_**stations**_**)**					**Length (cm) ± SD**					**Ambient temperature (°C)**				
	**Season ►**	**May**		**July**			**May**		**July**			**May**		**July**		
	**Water mass ▼**	**her**	**bwh**	**mac**	**her**	**bwh**	**her**	**bwh**	**mac**	**her**	**bwh**	**her**	**bwh**	**mac**	**her**	**bwh**
**2005**	**Arctic**	10 (1)	-	8 (1)	41 (5)	-	32.55 ± 0.93	-	41.00 ± 2.56	34.41 ± 1.36	-	2.84	-	9.30	9.30	-
	**Atlantic**	80 (8)	60 (6)	150 (15)	94 (10)	-	29.91 ± 3.64	24.09 ± 3.36	36.77 ± 3.22	32.59 ± 3.57	-	6.60	4.15	9.35	9.75	-
	**Coastal**	20 (2)	20 (2)	54 (6)	20 (2)	-	31.75 ± 1.11	20.95 ± 2.93	34.41 ± 3.54	30.03 ± 5.11	-	5.50	6.25	9.91	10.40	-
**2006**	**Arctic**	20 (2)	20 (2)	19 (2)	-	-	31.03 ± 2.45	24.25 ± 2.59	38.37 ± 1.83	-	-	4.25	4.62	8.29	-	-
	**Atlantic**	239 (24)	264 (28)	160 (16)	-	-	30.93 ± 2.45	25.43 ± 2.41	34.76 ± 3.73	-	-	5.08	4.95	10.94	-	-
	**Coastal**	40 (4)	29 (3)	50 (5)	-	-	29.90 ± 1.55	24.76 ± 1.88	34.24 ± 4.13	-	-	7.07	6.99	11.37	-	-
**2007**	**Arctic**	79 (8)	-	108 (11)	-	50 (7)	33.34 ± 2.05	-	37.84 ± 3.42	-	30.58 ± 2.01	2.25	-	6.98	-	7.89
	**Atlantic**	371 (38)	306 (32)	156 (16)	-	80 (8)	30.53 ± 2.42	27.06 ± 1.99	32.95 ± 3.99	-	27.98 ± 1.72	4.42	4.56	11.05	-	4.98
	**Coastal**	50 (5)	59 (6)	82 (9)	-	-	27.67 ± 3.31	25.43 ± 1.82	31.51 ± 4.57	-	-	6.29	5.51	12.97	-	-
**2008**	**Arctic**	-	-	-	15 (3)	-	-	-	-	30.73 ± 1.55	-	-	-	-	8.61	-
	**Atlantic**	113 (12)	103 (11)	-	25 (5)	-	29.83 ± 2.75	28.01 ± 1.53	-	30.24 ± 2.84	-	4.90	4.73	-	8.03	-
	**Coastal**	10 (1)	8 (1)	-	-	-	29.30 ± 1.25	27.19 ± 0.65	-	-	-	7.01	6.03	-	-	-
**2009**	**Arctic**	-	-	11 (2)	21 (4)	-	-	-	37.73 ± 4.78	33.43 ± 1.68	-	-	-	8.42	8.30	-
	**Atlantic**	90 (9)	20 (2)	50 (9)	37 (9)	-	32.13 ± 1.78	31.30 ± 1.52	35.28 ± 2.74	31.08 ± 2.45	-	3.92	4.59	11.35	8.88	-
	**Coastal**	10 (1)	10 (1)	10 (1)	4 (1)	-	29.20 ± 2.20	28.85 ± 1.58	32.90 ± 1.91	29.00 ± 3.44	-	4.02	4.02	12.74	12.81	-
**2010**	**Arctic**	10 (1)	-	79 (8)	155 (16)	-	34.25 ± 1.27	-	36.60 ± 1.83	34.93 ± 1.46	-	3.68	-	2.42	2.23	-
	**Atlantic**	19 (2)	10 (1)	390 (39)	197 (21)	77 (8)	31.21 ± 1.79	30.85 ± 1.06	34.97 ± 3.13	32.53 ± 1.74	31.06 ± 1.86	5.97	4.67	7.41	5.08	5.58
	**Coastal**	-	-	30 (3)	29 (3)	20 (2)	-	-	33.03 ± 3.27	29.95 ± 3.41	31.47 ± 1.70	-	-	8.28	7.39	4.30
**B**																
**Year**		**Condition Factor (CF) ± SD**					**Stomach Fullness Degree (SFD) ± SD** ^**(1)**^					**Feeding Incidence (FI) % ± SD**				
	**Season ►**	**May**		**July**			**May**		**July**			**May**		**July**		
	**Water mass ▼**	**her**	**bwh**	**mac**	**her**	**bwh**	**her**	**bwh**	**mac**	**her**	**bwh**	**her**	**bwh**	**mac**	**her**	**bwh**
**2005**	**Arctic**	0.70 ± 0.04	-	0.84 ± 0.04	0.89 ± 0.07	-	0.84 ± 0.69	-	1.20 ± 0.71	0.79 ± 0.61	-	100	-	100	100	-
	**Atlantic**	0.73 ± 0.05	0.56 ± 0.06	0.98 ± 0.09	0.90 ± 0.08	-	0.92 ± 1.46	0.59 ± 0.67	1.01 ± 0.99	0.87 ± 1.36	-	100	90.00 ± 20.00	93.33 ± 13.97	80.00 ± 26.67	-
	**Coastal**	0.69 ± 0.03	0.48 ± 0.03	0.99 ± 0.15	0.81 ± 0.05	-	0.54 ± 0.52	0.01 ± 0.02	1.25 ± 1.58	0.32 ± 0.14	-	100	15.00 ± 7.07	93.15 ± 12.18	100	-
**2006**	**Arctic**	0.70 ± 0.05	0.58 ± 0.14	0.99 ± 0.05	-	-	1.46 ± 1.92	1.05 ± 0.81	0.43 ± 0.29	-	-	100	100	90.00 ± 14.14	-	-
	**Atlantic**	0.71 ± 0.05	0.57 ± 0.05	1.01 ± 0.08	-	-	1.16 ± 1.40	0.44 ± 0.72	1.51 ± 0.51	-	-	97.50 ± 7.37	82.54 ± 28.08	91.25 ± 16.28	-	-
	**Coastal**	0.70 ± 0.05	0.57 ± 0.04	0.99 ± 0.11	-	-	0.52 ± 0.34	0.18 ± 0.36	1.58 ± 1.55	-	-	100	86.29 ± 5.48	100	-	-
**2007**	**Arctic**	0.76 ± 0.05	-	0.92 ± 0.08	-	0.55 ± 0.05	3.39 ± 2.91	-	1.19 ± 1.63	-	0.40 ± 0.86	100	-	89.77 ± 10.03	-	44.00 ± 8.94
	**Atlantic**	0.67 ± 0.04	0.57 ± 0.05	0.94 ± 0.09	-	0.56 ± 0.07	0.76 ± 0.85	0.73 ± 1.33	0.63 ± 0.62	-	0.85 ± 1.30	88.68 ± 22.08	83.06 ± 17.74	86.25 ± 21.87	-	78.75 ± 13.56
	**Coastal**	0.65 ± 0.04	0.55 ± 0.04	0.88 ± 0.10	-	-	0.52 ± 0.98	0.17 ± 0.25	0.83 ± 1.63	-	-	92.00 ± 8.37	43.89 ± 19.25	84.60 ± 14.93	-	-
**2008**	**Arctic**	-	-	-	0.85 ± 0.06	-	-	-	-	0.96 ± 1.70	-	-	-	-	100	-
	**Atlantic**	0.73 ± 0.05	0.60 ± 0.04	-	0.85 ± 0.06	-	1.45 ± 1.72	0.27 ± 0.34	-	0.56 ± 0.54	-	98.33 ± 5.77	89.78 ± 19.64	-	80.00 ± 24.49	-
	**Coastal**	0.72 ± 0.04	0.58 ± 0.04	-	-	-	0.59 ± 0.68	0.16 ± 0.24	-	-	-	100	100	-	-	-
**2009**	**Arctic**	-	-	0.87 ± 0.06	0.83 ± 0.09	-	-	-	1.38 ± 0.92	0.37 ± 0.32	-	-	-	90.00 ± 14.14	77.08 ± 31.46	-
	**Atlantic**	0.69 ± 0.04	0.67 ± 0.05	0.98 ± 0.08	0.88 ± 0.09	-	0.41 ± 0.32	2.53 ± 2.15	0.85 ± 1.30	0.38 ± 0.46	-	94.44 ± 16.67	95.00 ± 7.07	83.33 ± 29.15	75.56 ± 21.08	-
	**Coastal**	0.70 ± 0.08	0.59 ± 0.04	0.95 ± 0.07	0.89 ± 0.06	-	0.33 ± 0.19	0.15 ± 0.18	0.22 ± 0.27	1.66 ± 0.89	-	100	30	80	50	-
**2010**	**Arctic**	0.73 ± 0.03	-	0.88 ± 0.07	0.82 ± 0.06	-	1.97 ± 1.01	-	1.25 ± 1.11	1.41 ± 1.51	-	100	-	91.25 ± 16.42	100	-
	**Atlantic**	0.72 ± 0.05	0.66 ± 0.03	0.92 ± 0.08	0.89 ± 0.09	0.62 ± 0.08	0.27 ± 0.36	1.13 ± 0.88	0.83 ± 1.29	0.78 ± 0.83	0.48 ± 0.67	69.44 ± 27.49	90	84.87 ± 21.38	98.57 ± 3.59	91.25 ± 11.26
	**Coastal**	-	-	0.92 ± 0.08	0.89 ± 0.07	0.69 ± 0.09	-	-	0.57 ± 0.68	0.25 ± 0.30	0.89 ± 1.18	-	-			

N_fish_ and N_station_ denote the number of fish samples and sampling stations in each case, respectively. SD is the standard deviation. ^(1)^ SFD units: x10^-5^ mg mm^-1^. ‘mac’ mackerel; ‘her’ herring; ‘bwh’ blue whiting.

### Feeding-related variables

While stomach fullness degree (SFD) and feeding incidence (FI) provide information about current feeding conditions, the Condition factor (CF) provides information about recent feeding history (Table 2B). In mackerel, both SFD and FI, were positively influenced by decreasing temperature, in addition to a significant year effect (Table 3). The importance of temperature is also apparent when SFD and FI are compared across water masses (Table 2B). Only year as a random variable significantly affected the CF of mackerel (Table 3).

**Table 3:** **Parameters of models predicting (A) condition factor (CF), (B) stomach fullness degree (SFD) and (C) feeding incidence (FI) for mackerel, herring and blue whiting (see section 2.6 for model explanation)**.

**A**					
**species**	**model**	**Response variable: CF (Gaussian distribution of the error term)**			
**mackerel**	GLMM	*Random effect*	Intercept (SD)		
		year	3.70E-02		
		*Fixed effects*	Estimate	SE	p
		Intercept	0.95	0.02	***
**herring**	GLM	*Fixed effects*	Estimate	SE	p
		Intercept	0.71	1.38E-02	***
		year: 2006	-1.69E-02	1.35E-02	ns
		year: 2007	-4.21E-02	1.24E-02	***
		year: 2008	-1.07E-02	1.33E-02	ns
		year: 2009	-2.10E-02	1.26E-02	´
		year: 2010	-2.60E-03	1.42E-02	ns
		season	0.15	1.18E-02	***
		ambient T	3.69E-03	1.61E-03	*
**blue whiting**	GLMM	*Random effect*	Intercept (SD)		
		year	0.02		
		*Fixed effects*	Estimate	SE	p
		Intercept	0.32	0.05	***
		length	9.60E-03	1.60E-03	***
**B**					***
**species**	**model**	**Response variable: SFD**^**1/4**^ **(Gaussian distribution of the error term)**			
**mackerel**	GLMM	*Random effect*	Intercept (SD)		
		year	4.21E-02		
		*Fixed effects*	Estimate	SE	p
		Intercept	0.40	3.28E-02	***
		ambient T	-0.01	2.85E-03	***
**herring**	GLM	*Fixed effects*	Estimate	SE	p
		Intercept	-0.07	7.30E-02	ns
		length	0.01	2.37E-03	***
		season	-0.04	1.15E-02	**
**blue whiting**	GLMM	*Random effect*	Intercept (SD)		
		year	1.62E-06		
		*Fixed effects*	Estimate	SE	p
		Intercept	-0.25	8.27E-02	**
		length	0.02	3.14E-03	***
		season	-0.05	1.99E-02	**
**C**					
**species**	**model**	**Response variable: FI (Binomial distribution of the error term)**			
**mackerel**	GLMM	*Random effect*	Intercept (SD)		
		year	1.26		
		*Fixed effects*	Estimate	SE	p
		Intercept	8.04	2.20	**
		ambient T	-0.49	0.20	*
**herring**	GLMM	*Random effect*	Intercept (SD)		
		year	0.98		
		*Fixed effects*	Estimate	SE	p
		Intercept	11.46	2.73	***
		ambient T	-0.45	9.88E-02	***
		length	-0.20	7.88E-02	*
**blue whiting**	GLMM	*Random effect*	Intercept (SD)		
		year	0.44		
		*Fixed effects*	Estimate	SE	p
		Intercept	-5.75	2.04	*
		ambient T	-0.30	8.01E-02	**
		length	0.33	7.58E-02	***

‘length’: fish length (mm); ‘season’: May vs. July; ‘ambient T’: ambient temperature (at the maximum fishing depth). SD and SE represent the standard deviation and standard error, respectively. ´p < 0.1

*p < 0.05

**p < 0.01

***p < 0.001; ns, not significant.

The CF of herring also differed between years, but not in a random way as for mackerel (Table 3). Herring CF was also positively affected by higher ambient temperature, and was not surprisingly higher during the summer than in the spring. Again, the effect of temperature can be seen when Atlantic and Coastal water masses are compared with the colder Arctic water masses in Table 2. The SFD was significantly lower in the summer than in the spring, while it increased with increasing fish length; larger herring generally had fuller stomachs (Tables 2 and 3). On the other hand, greater lengths had a negative influence on FI. The FI was positively influenced by decreasing temperature, as it was for mackerel, and the year effect was significant (Table 3). In the cold Arctic water masses no empty herring stomachs were collected in May or July, except in July 2009 (Table 2B).

The CF for blue whiting was significantly influenced by year and was clearly positively influenced by fish length, with larger fish having higher CF (Tables 2 and 3). Larger blue whiting also had fuller stomachs, but SFD decreased from spring to summer and there was also a significant year effect (Table 3). As for both mackerel and herring, the FI for blue whiting was positively affected by lower temperatures. It also increased with increasing length and a year effect was apparent.

### Inter-specific differences: feeding-related variables

**Fig 3:**
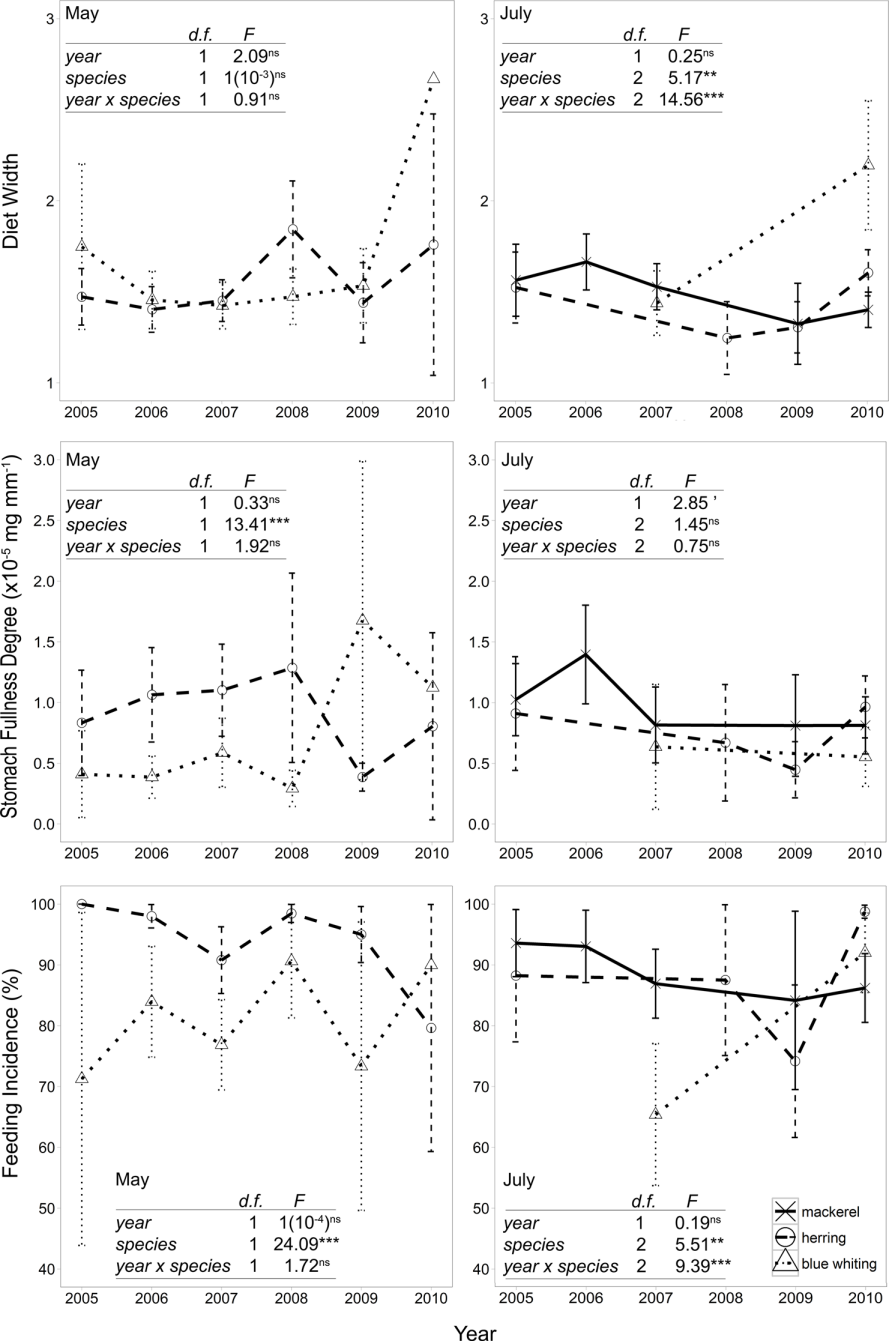
Average diet width, condition factor, stomach fullness and feeding incidence (± 2SE) for mackerel, herring and blue whiting, per year (from 2005 to 2010) and separated by seasons (May vs. July). The station and fish sample numbers are the same as in ‘Dataset 1’ (Table 1). Results from two-way ANOVAs (F tests for ‘year’, ‘species’ and ‘year x species’ interaction) for each response variable are indicated within each panel; d.f., degrees of freedom; ‘p < 0.1; *p < 0.05; **p < 0.01; ***p < 0.001; ns, not significant.

Overall, an average of between 1 and 3 different prey groups were consumed by blue whiting, which tended to consume a broader diet than herring. No significant inter-annual variation was observed in the diet width in any season (Fig 3). However, the diet width of blue whiting and herring was similar in May, while in July significant differences were observed, especially for blue whiting compared with the other species (Tukey HSD test, p < 0.001) and, to a lesser extent, between mackerel and herring (Tukey HSD test, p < 0.1) (Fig 3).

Herring and blue whiting showed different SFD in May, although there was no annual variation (Fig 3). In July, mackerel seemed to display a higher SFD, especially in 2006 (Fig 3), but no significant differences between the species were observed (Tukey HSD test, p > 0.1).

There was no annual variation on the feeding incidence in May or July, but there were significant inter-species differences (Fig 3). In May, herring showed a higher FI than herring, whereas in July blue whiting seemed to show lower values than the other species (Tukey HSD test: herring vs. blue whiting, p < 0.05; mackerel vs. blue whiting, p < 0.001; mackerel vs. herring, p < 0.05), except in 2010, when mackerel had the lowest value (Fig 3).

### General diet composition

**Fig 4:**
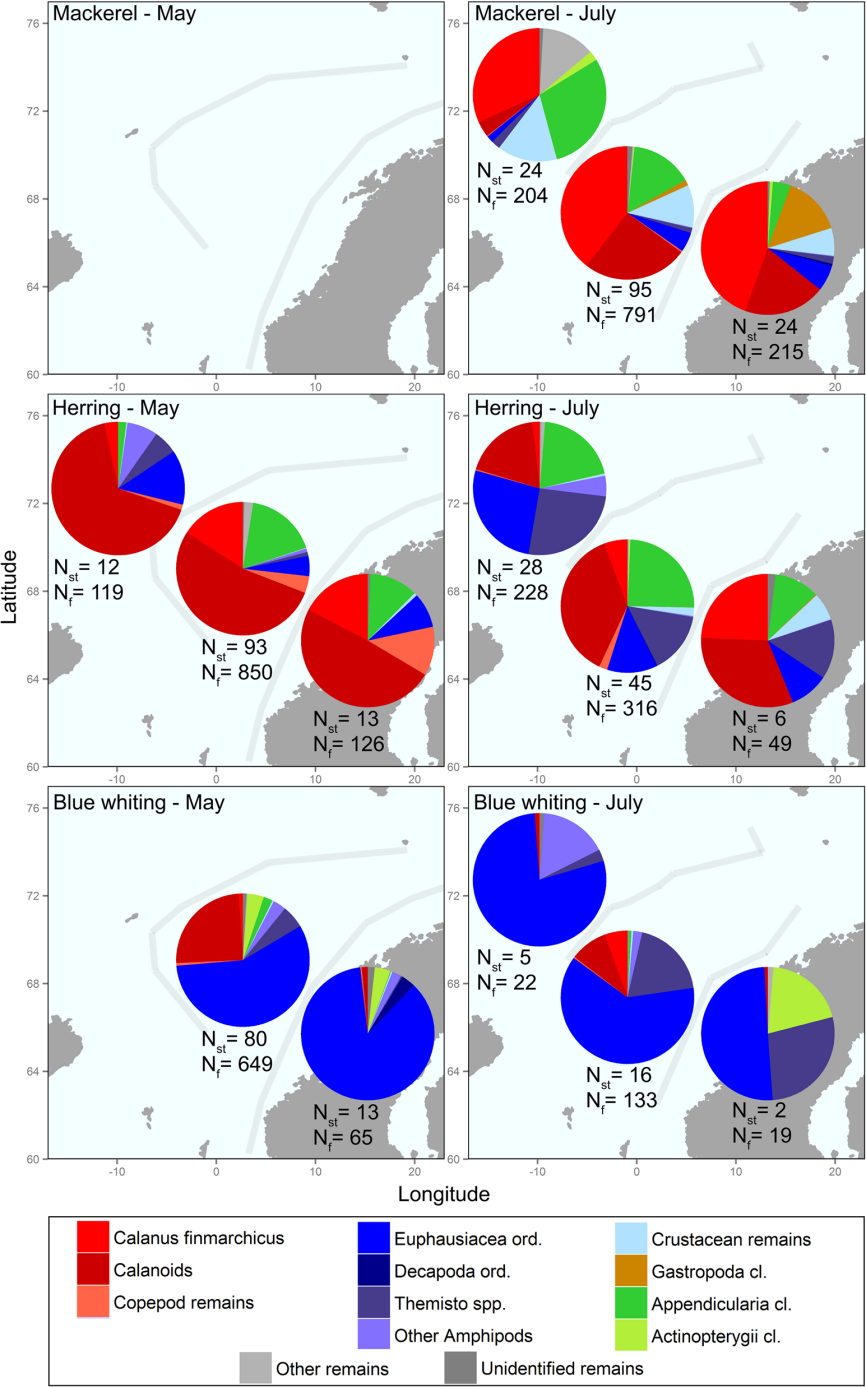
Average diet composition (mean prey group mg fish^-1^ weighted by the total estimated abundance per station), in percentages, for mackerel, herring and blue whiting in different water masses in May and July. All sampling years were analysed together (i.e. ‘Dataset1’ in Table 1; all stations in Fig 1). Light grey lines represent the average water mass boundaries during each season. N_st_ and N_f_ are the number of stations and fish samples, respectively. Empty stomachs were excluded from this analysis.

When diet data from all water masses and years are considered, mackerel and herring had similar diet compositions, with calanoid copepods (especially *C*. *finmarchicus*) as the dominant prey item. The ingestion of other groups like appendicularians and euphausiids (which were particularly abundant in herring stomachs in July) was also pronounced in some seasons and years. The blue whiting diet included more larger prey like euphausiids and amphipods (e.g. *Themisto* spp.), and less copepods than the diet of the two other species (Fig 4).

The ingestion of appendicularians by mackerel was especially important in Arctic waters, while the euphausiids were more abundant in coastal waters. In Atlantic waters, the mackerel diet mainly comprised copepods (*C*. *finmarchicus*). For herring, copepods (e.g. *C*. *finmarchicus*), were especially abundant as prey in May, while these were partially replaced by euphausiids and amphipods in July. The seasonal difference in diet was most obvious in the coastal water mass. No seasonal or water mass differences were observed for the ingestion of appendicularians. The amount of copepods ingested by blue whiting was generally low and limited to the Atlantic water mass. Larger euphausiids and/or amphipods dominated blue whiting diet in all water masses and seasons, and fish were also found in the stomachs from two coastal stations in July (Fig 4).

### Diet composition of pelagic planktivorous species co-occurring in the Atlantic water mass

**Fig 5:**
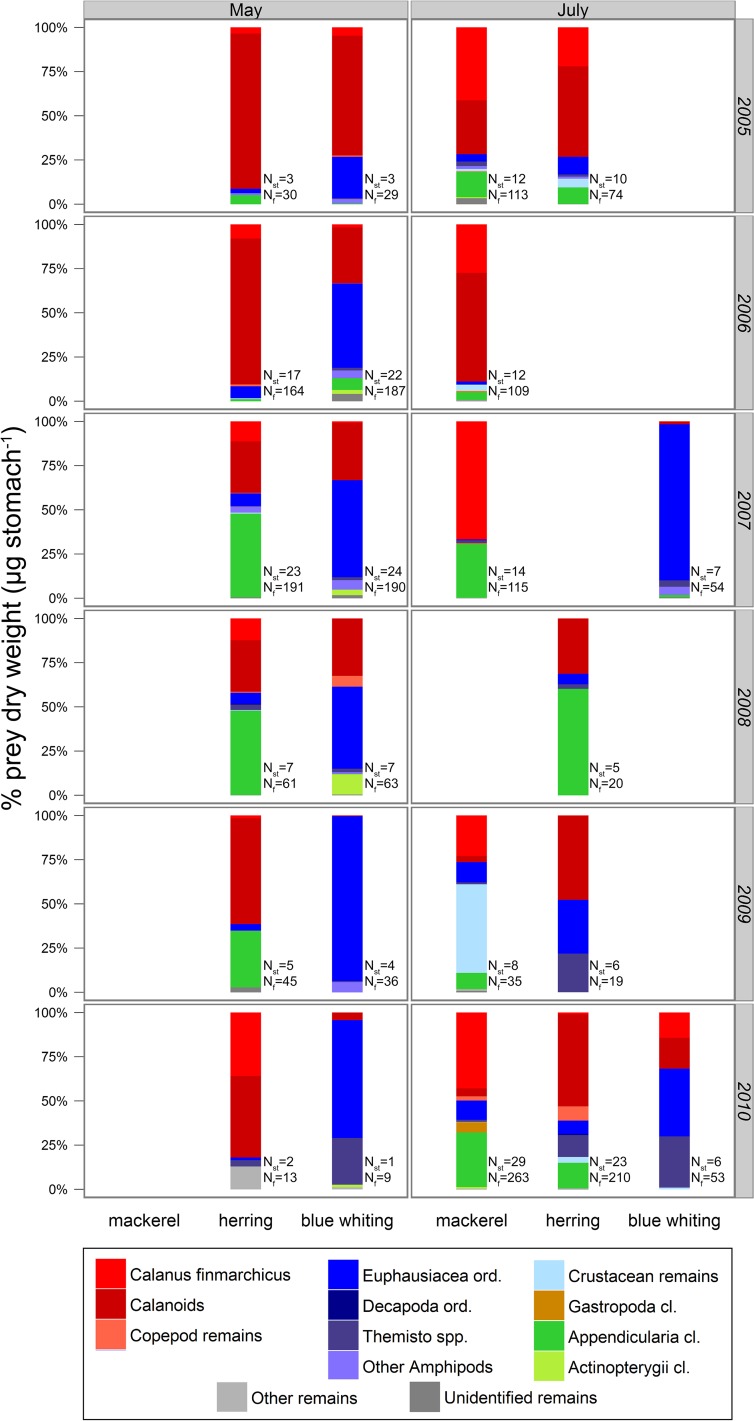
Average diet composition (mean prey group mg fish^-1^ weighted by total estimated abundance per station), in percentages, for mackerel, herring and blue whiting, per year (i.e., from 2005 to 2010) and season (i.e., May and July). Stations included in this analysis were those within the Atlantic water mass and with spatial overlap between ≥2 predator species (‘Dataset2’, Table 1). Prey group categorisation was simplified into 13 groups (see section 2.5). N_st_ and N_f_ are the number of stations and fish samples, respectively. The detailed diet characterization corresponding to each case (i.e. based on 45 prey group categorisations) is presented in Table A.1. Empty stomachs were excluded from this analysis.

When the diet composition restricted to the Atlantic water masses with co-occurrence of at least two of the three species in the samples is analysed, mackerel had a calanoid (*C*. *finmarchicus*) copepod-based diet in almost all the years of sampling. However, other prey groups were also present in the diet, especially from 2007 to 2010, when the copepod contribution was partially replaced by other crustaceans and appendicularians (Fig 5). The herring diet in May was also largely based on calanoid copepods (*C*. *finmarchicus*), and this was most pronounced in 2005 and 2006. From 2007–2009, other groups, such as appendicularians and euphausiids, were also important prey items. In 2010, calanoid copepods were again the dominant prey group. Large prey (typically appendicularians, euphausiids and amphipods) tended to occur more frequently in the diet in July than in May (Fig 5). Blue whiting diet was clearly different from the diet of the other two species. With the exception of May 2005, when copepods appeared as dominant prey items, euphausiids and amphipods dominated the diet both in May and July. Amphipods in particular were more common in the blue whiting diet than the other diets. Some ingestion of large prey was also detected (e.g. fish of order Actinopterygii in May 2008). Copepod contribution in the blue whiting diet for May decreased during the last sampling years (2009–2010) (Fig 5).

A detailed diet characterization for all species categorised into season and year is presented as supplementary material (S1 Table).

### Dietary overlap in the Atlantic water mass

The diet overlap (*O*) was tested separately for stations with and without spatial co-occurrence between the species compared within the Atlantic water mass (Table 4). The dietary overlap between herring and blue whiting was lower than expected by chance for all comparisons, except for July without co-occurrence. Also between mackerel and blue whiting, the dietary overlap was lower than expected by chance both with and without co-occurrence. Mackerel and herring had a higher dietary overlap when they co-occurred (0.77), but lower than would have been expected by chance without co-occurrence (Table 4).

**Table 4:** Pianka’s index of niche overlap (*O*) of paired comparisons between the three pelagic planktivores for May and July. Comparisons are made separately for stations with and without sample co-occurrence of the two species compared, considering all the stations and years combined.

predsp1	predsp2	co-occurrence	May		July	
			*O*	p (obs ≤ exp)	*O*	p (obs ≤ exp)
mackerel	herring	yes	-		0.77	ns
mackerel	blue whiting	yes	-		0.63	*
herring	blue whiting	yes	0.63	*	0.48	**
mackerel	herring	no	-		0.64	*
mackerel	blue whiting	no	-		0.55	**
herring	blue whiting	no	0.66	*	0.63	ns

‘predsp1’: predator species 1; ‘predsp2’: predator species 2. Overlap indices were calculated on the basis of the five merged prey-group characterization (see section 2.6). The probability (p) of observed (obs) vs expected (exp) overlap is based on 1000 simulated replicates:

*p (obs ≤ exp) < 0.1

**p (obs ≤ exp) < 0.01; ns, not significant. In each replicate the observed proportion of a diet group utilised by a species is replaced by a uniform value between 0 and 1, except zero utilisation values, which are retained (see section 2.5 for details).

**Fig 6:**
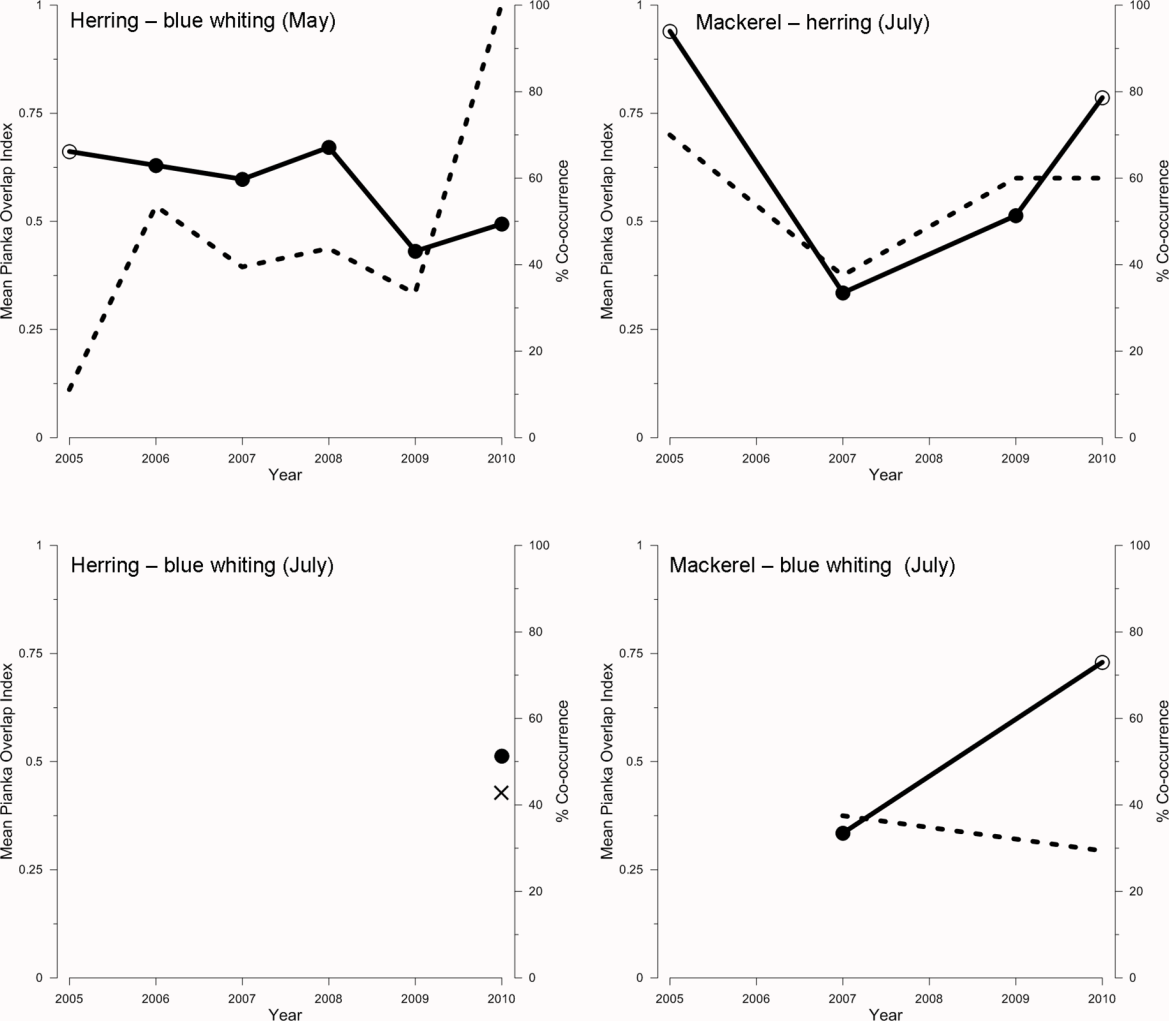
Mean Pianka index of overlap in diet and percentage of co-occurrence between different predator species (paired comparisons), in May and July from 2005 to 2010. The left axes and continuous lines represent the overlap, and the right axes and dotted lines represent the percentage of co-occurrence between species. In the case of the overlap index, filled symbols indicate that the probability of observed overlap is significantly lower than expected (based on 1000 simulated replicates), i.e., p(obs ≤ exp) < 0.05 (see section 2.5 for details). For herring–blue whiting (July) panel (lower left), the single co-occurrence measurement is indicated with a cross.

There was little variation in the fraction of stations with co-occurrence of herring and blue whiting between 2006 and 2009 (45–55%) (Fig 6). In 2005 and 2010, the minimum and maximum rates of co-occurrence were observed, respectively, while dietary overlap did not display major differences from the other years. In July, the number of stations with co-occurrence of mackerel and herring fitted well with the degree of dietary overlap, suggesting that higher co-occurrence could result in higher dietary overlap between these species. In contrast, blue whiting seemed to show an opposite relationship between co-occurrence and dietary overlap with mackerel, suggesting a lower overlap when they shared an ecological niche. Unfortunately, the limited number of stations with blue whiting in July (only caught together with herring in 2010) did not allow further comparisons in earlier years to be made (Fig 6).

## Discussion

### Water mass boundaries and prey distribution

The water mass boundaries, which defined a framework for several of our analyses, were similar in May and July, but the southern limit of the Arctic boundary was more restricted to south-eastern Icelandic waters in July. The boundaries defined in the present study are similar to those described in the 1980s by Johannessen [35] and Blindheim [36], and also to those defined in later studies covering the same geographical regions, e.g. Broms et al. [24] in the 1990s; Dalpadado et al. [3] and Langøy et al. [16] in the 2000s.

Zooplankton were patchily distributed all over the NS both in May and July, and zooplankton <2000 μm was the most abundant fraction all over the NS, especially in Atlantic water masses. A large proportion of this size fraction is probably made up of *C*. *finmarchicus*, which dominates the zooplankton species in this area [8,15,16,22,37]. The largest size range of zooplankton is typically dominated by euphausiids (*Meganyctiphanes norvegica*, *Thysanoessa inermis*, *T*. *longicaudata*) and amphipods (*Themisto libellula*), which are widely distributed over the sampling area [3,8,37]. These macrozooplankton are not representatively sampled with WP2 plankton nets [3,36–38], but the relatively higher biomass of this large group found in Arctic waters is probably still indicative of a difference in plankton communities between the water masses, which is in accordance with results from previous studies [3,19,36]. On the other hand, the small and slightly significant reduction in mid-sized zooplankton (the >1000μm fraction, corresponding to *C*. *finmarchicus*) from May to July at least within the Atlantic water mass might partially reflect the end of the peak abundance of *C*. *finmarchicus* [3,14,22,24,37]. This might suggest that some kind of relationship exists between the distribution of zooplankton and that of planktivorous fish [37], resulting in a depletion of the copepod population, probably affected by the major feeding activity especially of herring (feeding mainly in spring–early summer) and mackerel (feeding mainly during the summer) in the NS ([3,13],this study)]. However, in the Atlantic water mass, the smallest organisms remained in large quantities in July, and moreover, such depletion was not reflected in our results when the total biomass distribution was observed. That suggests that prey availability does not change much from May to July, nor between the sampling years, despite the fact that the total available zooplankton biomass appears to have been higher in 2010 than in previous years.

### Fish distribution

Due to the annual and seasonal variations in sampling effort in our study, only limited inferences about the real distribution of the three planktivorous species can be made. For instance, it is obvious that the surface trawling employed in July surveys (Table 1) is not adequate for representative sampling of blue whiting, which are typically distributed in deeper waters [18,19,39], although they may perform diel vertical migrations to the surface. Despite these shortcomings, it seems clear that mackerel were not present in the NS in May during our study period, even though they have been observed in these areas in May very recently [13]. The absence of mackerel in the spring is in accordance with observations from previous studies of mackerel peak feeding in late summer [7,17]. It is also clear from our results that the herring distribution in the NS in May was very wide, while the true distribution in July was probably not well reflected in the acoustic data, due to a shallow distribution of the species above the echo-sounder’s detection range [9].

### Diet composition and feeding

Calanoid copepods dominated the diets of mackerel and herring, which is in agreement with the results of several earlier studies [3,15,16,20,40]. Calanoid copepods (mainly *C*. *finmarchicus*) are also by far the most abundant prey group in the Atlantic water masses of the NS [4,8,14,37]. Several authors have linked the annual distribution and migration patterns of herring to the life cycle of *C*. *finmarchicus* (e.g. [5,22,24]), and the peak feeding in May is accordingly reflecting the peak abundance of *C*. *finmarchicus*. Our results support this premise: firstly, since there was a higher proportion of other and typically larger prey than calanoids in July than in May; secondly, since herring SFD was higher in May than in July; and thirdly, since CF for herring was clearly higher in July than in May, likely reflecting high feeding intensity during spring and early summer [16]. However, even though the FI of herring was slightly lower in July than in May, in accordance with Langøy et al. [16] and Prokopchuck and Sentyabov [15], the herring SFD was not lower than that of mackerel in July, indicating that herring also feed heavily in summer. SFD also increased with herring size, suggesting that large herring feed more effectively, whereas larger herring also had lower FI, indicating that they search for prey abundant areas, or else cease to feed, in order to compensate their energetic demands. This is in accordance with our result that FI increased with decreasing temperature, and that both mackerel and herring ingested less copepods and more euphausiids and amphipods in Arctic water masses than in the other regions, suggesting that they expand their distribution further north-east following larger prey. The results are also consistent with the normally longer migrations to plankton-rich areas observed for larger herring [3]. Furthermore, in relation to FI and SFD, mackerel had higher scores than the other two species, in accordance with previous studies demonstrating the voracity of feeding mackerel [41,42].

Our results demonstrated the importance of annual effects when the diet composition is under consideration. While the herring diet in May mainly consisted of calanoids (*C*. *finmarchicus*), copepods were partially replaced by appendicularians and euphausiids from 2007 to 2009. Interestingly, appendicularians were important prey for both mackerel and herring during certain seasons and years. This supports the results of Langøy et al. [16] and Prokopchuck & Sentyabov [15], but the importance of appendicularians was even more pronounced in our study, and seems to rule out the possibility that these are coincidental prey items swallowed during search or filter feeding. Both mackerel and herring are able to switch from filter feeding to opportunistic particulate feeding when larger prey are available [43,44], and according to Purcell et al. [45], the great abundance of appendicularians, their high nutritional value and their slow swimming speed and lack of carapace make them attractive as prey for many predators. We have limited information about the spatial distribution of appendicularians, which were found in stomach contents in large numbers but only at certain stations, indicating that they are locally important prey items with a patchy distribution [16].

Blue whiting were found to have a different diet composition than herring and mackerel, with more ingestion of relatively large prey like euphausiids (e.g. *M*. *norvegica*) and particularly amphipods (e.g. *T*. *libellula*) during May and July. This result is in agreement with previous studies [15,16], and supports the notion that blue whiting to a large extent are adapted to a different niche than herring and mackerel, feeding more on amphipods and euphausiids [15,16,45] in deeper waters, where such larger prey might be found in greater abundance [14], and under more homogeneous temperature regimes [18,19,39]. Moreover, our results indicate that blue whiting can effectively use a wide range of prey sizes, including the most abundant and patchily distributed smaller zooplankton fractions [15], and hence in general enjoy a broader diet than the other two species. The higher SFD and FI with increasing length of blue whiting could also indicate a higher success in predation upon large prey (i.e. lower allometric limitations due to size [44]). This is also in accordance with the positive relation between the CF and the length of blue whiting, and could indicate that large fish have been more successful in predation upon large prey than small fish, compensating their higher energetic demands in summer when they show lower SFD and the small prey availability is lower.

### Co-occurrence and dietary overlap

The highest dietary overlap in our study was between herring and mackerel, and it was higher when they co-occurred (were caught in the same sample), than otherwise. This suggests that they were largely feeding on the same patches of food when they co-occurred, in accordance with an opportunistic and dynamic feeding behaviour [13,16,28,44]. On the basis of samples from all the stations combined, mackerel appear to feed most extensively on copepods, but considering only the stations with co-occurrence of herring and mackerel, the percentage of copepods in the diet is highest for herring, both in May and July. Furthermore, in July 2009–2010, when the seasonal difference in the herring diet was clearest, mackerel also incorporated other prey than copepods in their diet (e.g. gastropods, fish, other crustaceans and appendicularians) likely due to reduced copepod availability. Together, these results suggest that strong competition leading to prey switch from one species does not occur between herring and mackerel, which concurs with the results of the two-year study for summer 2001 and 2002 by Prokopchuck and Sentyabov [15], and for summer 2004 and 2006 by Langøy et al. [16].

Blue whiting showed lower dietary overlap with herring in co-occurring stations than in those stations without co-occurrence in both May and July. This suggests that they are able to avoid competition by feeding on different prey than herring when the two species co-occur.

## Concluding Remarks

Our results, based on diet data of mackerel, herring and blue whiting acquired during spring and summer 2005–2010, showed that mackerel and herring diets largely overlapped, with calanoid copepods being their main prey item, while the blue whiting diet consisted of larger prey items, particularly amphipods. Mackerel were not present in the study area in spring, and seemed to show good feeding conditions and predation success in the NS during summer. Herring showed their highest feeding incidence during the spring. However, contrary to expectations, herring showed a similar degree of stomach fullness as mackerel in summer, but with a diet composed more of larger macrozooplankton rather than copepods, indicating that in particular larger herring adopt a strategy of migrating into cold waters and search for larger prey. Blue whiting seemed to avoid competition with co-occurring herring by feeding on different prey, and the higher stomach fullness degree, feeding incidence and condition factor observed for the largest blue whiting suggested a higher feeding success than that observed for small blue whiting.

In addition, there were clear annual effects on diet composition and feeding variables, which demonstrates the ability of these species to adapt their feeding to different conditions. For instance were appendicularians important prey items in certain seasons and years. The ability of small pelagic species to adapt their diet composition to a wide prey size range and diversity is important to take into account when considering the foraging pressure and carrying capacity of the NS ecosystem, and has also been observed in other ecosystems (e.g. the Bay of Biscay, [28,44]). As an illustration of a possible effect, the combined influence of high abundance and thus of extensive copepod consumption of mackerel early in the season in the NS, as has been observed in recent years [9,11,13], together with large fish size with a greater migratory capacity, will likely lead to more feeding in the Arctic waters, where the plankton community is characterised by larger species, fish show more active feeding and the diet composition is similar for all three planktivorous species. In turn, this is likely to lead to more competition and higher predation pressure on the standing stocks of larger zooplankton.

Further research should focus on comparing vertical and horizontal spatial overlap with the dietary overlap, and on estimating the consumption of different prey groups by predator species. This would be of great value as input for operational ecosystem-based approaches to stock management.

## Supporting Information

S1 Fig Zooplankton total biomass (dry weight, g m^-2^) distribution per year (2005–2010) in May and July. Dark grey lines indicate water mass boundaries for each year and season, and light grey lines represent the average boundaries during each season.
(TIF)

S2 Fig **Estimated fish abundance distribution per year (2005–2010) in May and July for (A) mackerel (July), (B) herring and (C) blue whiting.** Abundances per grid square (see section 2.3) are defined as thousands of tonnes for herring and blue whiting, and in CPUE for mackerel. Black symbols represent sampling stations. Dark grey lines indicate water-mass boundaries for each year and season, and light grey lines represent the average boundaries during each season.
(TIF)

S1 Table Average prey composition in percentages (mean mg fish^-1^ weighed with the total estimated abundance per station) for mackerel, herring and blue whiting in spring and summer and from 2005 to 2010, based on the highest taxonomic level categorization (i.e. 45 prey groups). Stations included in this analysis were those within the Atlantic water mass and with spatial overlap of ≥2 predator species (‘Dataset2’, Table 1). N_stations_ and N_fish_ are the number of stations and fish samples respectively. ‘spr’: spring (May survey); ‘smr’: summer (July survey).
(DOC)
