# Supplementary figures and images for: Feeding Ecology of Northeast Atlantic Mackerel, Norwegian Spring-Spawning Herring and Blue Whiting in the Norwegian Sea
Source: PLoS One. 2016 Feb 19;11(2):e0149238. doi: 10.1371/journal.pone.0149238 (PMC4764516; doi:10.1371/journal.pone.0149238)

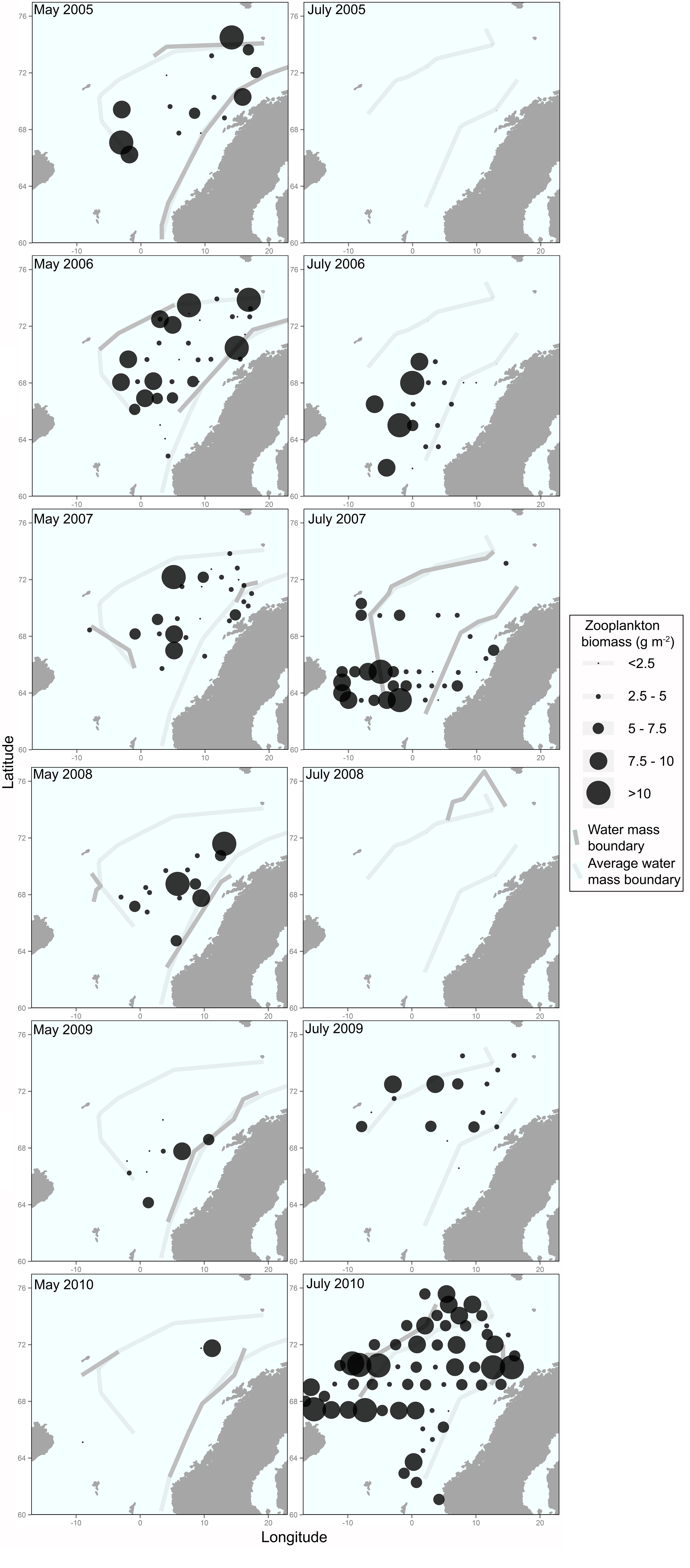

Supplement: S1 Fig — Dark grey lines indicate water mass boundaries for each year and season, and light grey lines represent the average boundaries during each season. (TIF) [file pone.0149238.s001.tif]
