# Supplementary material for: Feeding Ecology of Northeast Atlantic Mackerel, Norwegian Spring-Spawning Herring and Blue Whiting in the Norwegian Sea
Source: PLoS One. 2016 Feb 19;11(2):e0149238. doi: 10.1371/journal.pone.0149238 (PMC4764516; doi:10.1371/journal.pone.0149238)
Supplement: S1 Table — Stations included in this analysis were those within the Atlantic water mass and with spatial overlap of ≥2 predator species (‘Dataset2’, Table 1). Nstations and Nfish are the number of stations and fish samples respectively. ‘spr’: spring (May survey); ‘smr’: summer (July survey). (DOC) [file pone.0149238.s003.doc]

[Supplementary Tables]

**S1 Table** **Average prey composition in percentages (mean mg fish^-1^ weighed with the total estimated abundance per station) for mackerel, herring and blue whiting in spring and summer and from 2005 to 2010, based on the highest taxonomic level categorization (i.e. 45 prey groups)**. Stations included in this analysis were those within the Atlantic water mass and with spatial overlap of ≥2 predator species (‘Dataset2’, Table 1). N_stations_ and N_fish_ are the number of stations and fish samples respectively. ‘spr’: spring (May survey); ‘smr’: summer (July survey).

| **Year** |  | **2005** | | | | | | | |  | **2006** | | | | | | | |
| --- | --- | --- | --- | --- | --- | --- | --- | --- | --- | --- | --- | --- | --- | --- | --- | --- | --- | --- |
| **Species** |  | **Mackerel** | |  | **Herring** | |  | **Blue whiting** | |  | **Mackerel** | |  | **Herring** | |  | **Blue whiting** | |
| **Month (season)** |  | **May** | **July** |  | **May** | **July** |  | **May** | **July** |  | **May** | **July** |  | **May** | **July** |  | **May** | **July** |
| **N_stations_** |  | 0 | 12 |  | 3 | 10 |  | 3 | 0 |  | 0 | 12 |  | 17 | 0 |  | 22 | 0 |
| **N_fish_** |  | 0 | 113 |  | 30 | 74 |  | 29 | 0 |  | 0 | 109 |  | 164 | 0 |  | 187 | 0 |
| *Calanus finmarchicus* |  | - | 41.24 |  | 3.40 | 21.88 |  | 4.56 | - |  | - | 27.39 |  | 7.90 | - |  | 1.66 | - |
| *Calanus glacialis* |  | - | 0 |  | 0.13 | 0 |  | 1.46 | - |  | - | 3.04E-08 |  | 0.47 | - |  | 0.02 | - |
| *Calanus hyperboreus* |  | - | 0 |  | 2.84 | 0 |  | 17.07 | - |  | - | 0 |  | 6.43 | - |  | 7.07 | - |
| *Calanus* spp. |  | - | 29.81 |  | 84.81 | 2.01 |  | 48.69 | - |  | - | 7.23 |  | 74.42 | - |  | 24.39 | - |
| *Clausocalanus* spp. |  | - | 0 |  | 0 | 0 |  | 0 | - |  | - | 0 |  | 0 | - |  | 2.12E-06 | - |
| *Euchaeta* spp. |  | - | 0.57 |  | 0 | 0 |  | 0 | - |  | - | 0 |  | 0 | - |  | 2.10E-05 | - |
| *Metridia longa* |  | - | 0 |  | 0.05 | 0 |  | 0.66 | - |  | - | 1.59E-03 |  | 0.49 | - |  | 2.15E-06 | - |
| *Metridia* spp. |  | - | 0 |  | 0 | 2.77 |  | 0 | - |  | - | 0.06 |  | 0.80 | - |  | 4.61E-03 | - |
| *Calanoida ord. |  | - | 0.03 |  | 0 | 46.45 |  | 0 | - |  | - | 54.19 |  | 0 | - |  | 0 | - |
| *Copepoda subcl. |  | - | 0 |  | 0 | 0 |  | 0.67 | - |  | - | 0 |  | 1.06 | - |  | 0.12 | - |
| **Total copepods** |  | - | ***71.66*** |  | ***91.23*** | ***73.12*** |  | ***73.10*** | - |  | - | ***88.87*** |  | ***91.57*** | - |  | ***33.27*** | - |
| *Meganyctiphanes norvegica* |  | - | 0.05 |  | 0 | 0 |  | 0.72 | - |  | - | 0 |  | 0 | - |  | 1.38 | - |
| *Thysanoessa inermis* |  | - | 0 |  | 0 | 0 |  | 0 | - |  | - | 0 |  | 0 | - |  | 0 | - |
| *Thysanoessa longicaudata* |  | - | 2.57E-03 |  | 0 | 0.13 |  | 0 | - |  | - | 0 |  | 0 | - |  | 0 | - |
| *Thysanoessa* spp. |  | - | 0 |  | 0 | 0 |  | 0 | - |  | - | 0 |  | 0 | - |  | 0 | - |
| *Euphausiacea ord. |  | - | 4.13 |  | 2.55 | 9.89 |  | 22.75 | - |  | - | 1.52 |  | 6.61 | - |  | 46.44 | - |
| **Total euphausiids** |  | - | ***4.18*** |  | ***2.55*** | ***10.02*** |  | ***23.47*** | - |  | - | ***1.52*** |  | ***6.61*** | - |  | ***47.82*** | - |
| *Pandalus* spp. |  | - | 0 |  | 0 | 0 |  | 0 | - |  | - | 0 |  | 0 | - |  | 0 | - |
| *Caridea infraord. |  | - | 0 |  | 0 | 0 |  | 0 | - |  | - | 0 |  | 0 | - |  | 0 | - |
| *Decapoda ord. |  | - | 0 |  | 0 | 0 |  | 0 | - |  | - | 0.02 |  | 0 | - |  | 0 | - |
| **Total decapods** |  | - | ***0.00*** |  | ***0.00*** | ***0.00*** |  | ***0.00*** | - |  | - | ***0.02*** |  | ***0.00*** | - |  | ***0.00*** | - |
| *Themisto abyssorum* |  | - | 2.53 |  | 0 | 1.10 |  | 0.41 | - |  | - | 0.04 |  | 0.02 | - |  | 1.29 | - |
| *Themisto libellula* |  | - | 0.04 |  | 0 | 0 |  | 0 | - |  | - | 0 |  | 0 | - |  | 0.23 | - |
| *Themisto* spp. |  | - | 0.01 |  | 0 | 0.14 |  | 0 | - |  | - | 0.19 |  | 0 | - |  | 0 | - |
| *Hyperiidae fam. |  | - | 0 |  | 0 | 0 |  | 0 | - |  | - | 0 |  | 0 | - |  | 0 | - |
| *Amphipoda ord. |  | - | 1.51 |  | 0.98 | 1.11 |  | 2.63 | - |  | - | 1.11E-03 |  | 0.17 | - |  | 4.24 | - |
| **Total amphipods** |  | - | ***4.10*** |  | ***0.98*** | ***2.36*** |  | ***3.04*** | - |  | - | ***0.24*** |  | ***0.19*** | - |  | ***5.76*** | - |
| *Evadne* spp. |  | - | 0 |  | 0 | 0 |  | 0 | - |  | - | 2.03E-03 |  | 0 | - |  | 0 | - |
| *Podon* spp. |  | - | 0 |  | 0 | 0 |  | 0 | - |  | - | 0 |  | 0 | - |  | 0 | - |
| *Cladocera infraord. |  | - | 0 |  | 0 | 0 |  | 0 | - |  | - | 0 |  | 0 | - |  | 0 | - |
| **Total cladocerans** |  | - | ***0.00*** |  | ***0.00*** | ***0.00*** |  | ***0.00*** | - |  | - | ***0.00*** |  | ***0.00*** | - |  | ***0.00*** | - |
| Ostracoda cl. |  | - | 0 |  | 0 | 0 |  | 0 | - |  | - | 0 |  | 0 | - |  | 0 | - |
| *Crustacea subph. |  | - | 1.46 |  | 0 | 4.98 |  | 0 | - |  | - | 3.55 |  | 0.48 | - |  | 0 | - |
|  |  |  |  |  |  |  |  |  |  |  |  |  |  |  |  |  |  |  |
| **Total crustaceans** |  | - | ***81.40*** |  | ***94.76*** | ***90.48*** |  | ***99.61*** | - |  | - | ***94.20*** |  | ***98.85*** | - |  | ***86.85*** | - |
|  |  |  |  |  |  |  |  |  |  |  |  |  |  |  |  |  |  |  |
| *Gonatus fabricii* |  | - | 0 |  | 0 | 0 |  | 0 | - |  | - | 0.20 |  | 0 | - |  | 0 | - |
| *Cephalopoda cl. |  | - | 0 |  | 0 | 0 |  | 0 | - |  | - | 0.15 |  | 0.00 | - |  | 0.05 | - |
| **Total cephalopods** |  | - | ***0.00*** |  | ***0.00*** | ***0.00*** |  | ***0.00*** | - |  | - | ***0.36*** |  | ***0.00*** | - |  | ***0.05*** | - |
| *Limacina retroversa* |  | - | 6.64E-07 |  | 0 | 0.03 |  | 0 | - |  | - | 0.71 |  | 0.01 | - |  | 0 | - |
| *Limacina* spp. |  | - | 0.37 |  | 0 | 1.76E-06 |  | 0 | - |  | - | 0.02 |  | 0 | - |  | 0 | - |
| *Gastropoda cl. |  | - | 0 |  | 0 | 0 |  | 0 | - |  | - | 6.62E-03 |  | 0 | - |  | 0 | - |
| **Total gastropods** |  | ***-*** | ***0.37*** |  | ***0.00*** | ***0.03*** |  | ***0.00*** | ***-*** |  | ***-*** | ***0.74*** |  | ***0.01*** | ***-*** |  | ***0.00*** | ***-*** |
| Chaetognatha ph. |  | - | 1.07E-07 |  | 0 | 0 |  | 0 | - |  | - | 0.05 |  | 0 | - |  | 0 | - |
| Hydrozoa cl. |  | - | 0 |  | 0 | 0 |  | 0 | - |  | - | 0.02 |  | 0 | - |  | 0 | - |
|  |  |  |  |  |  |  |  |  |  |  |  |  |  |  |  |  |  |  |
| **Total invertebrates** |  | ***-*** | ***81.77*** |  | ***94.76*** | ***90.51*** |  | ***99.61*** | ***-*** |  | ***-*** | ***95.37*** |  | ***98.86*** | ***-*** |  | ***86.90*** | ***-*** |
|  |  |  |  |  |  |  |  |  |  |  |  |  |  |  |  |  |  |  |
| *Oikopleura* spp. |  | - | 0 |  | 0 | 0 |  | 0 | - |  | - | 0 |  | 0 | - |  | 0 | - |
| *Appendicularia cl. |  | - | 14.20 |  | 5.23 | 9.38 |  | 0.39 | - |  | - | 4.26 |  | 1.09 | - |  | 6.80 | - |
| **Total tunicates** |  | ***-*** | ***14.20*** |  | ***5.23*** | ***9.38*** |  | ***0.39*** | ***-*** |  | ***-*** | ***4.26*** |  | ***1.09*** | ***-*** |  | ***6.80*** | ***-*** |
| Myctophidae fam. |  | - | 0 |  | 0 | 0 |  | 0 | - |  | - | 0 |  | 0 | - |  | 0 | - |
| *Actinopterygii cl. |  | - | 0.63 |  | 0 | 0 |  | 0 | - |  | - | 4.05E-04 |  | 0 | - |  | 2.11 | - |
| **Total teleostei** |  | ***-*** | ***0.63*** |  | ***0.00*** | ***0.00*** |  | ***0.00*** | ***-*** |  | ***-*** | ***0.00*** |  | ***0.00*** | ***-*** |  | ***2.11*** | ***-*** |
|  |  |  |  |  |  |  |  |  |  |  |  |  |  |  |  |  |  |  |
| **Total vertebrates** |  | ***-*** | ***14.83*** |  | ***5.23*** | ***9.38*** |  | ***0.39*** | ***-*** |  | ***-*** | ***4.26*** |  | ***1.09*** | ***-*** |  | ***8.91*** | ***-*** |
|  |  |  |  |  |  |  |  |  |  |  |  |  |  |  |  |  |  |  |
| Other remains |  | - | 0 |  | 0 | 0 |  | 0 | - |  | - | 0 |  | 0 | - |  | 0 | - |
| Unidentified remains |  | - | 3.40 |  | 0.01 | 0.11 |  | 0 | - |  | - | 0.38 |  | 0.05 | - |  | 4.20 | - |
|  |  |  |  |  |  |  |  |  |  |  |  |  |  |  |  |  |  |  |
| **Total** |  | ***-*** | ***100.00*** |  | ***100.00*** | ***100.00*** |  | ***100.00*** | ***-*** |  | ***-*** | ***100.00*** |  | ***100.00*** | ***-*** |  | ***100.00*** | ***-*** |

| **Year** |  | **2007** | | | | | | | |  | **2008** | | | | | | | |
| --- | --- | --- | --- | --- | --- | --- | --- | --- | --- | --- | --- | --- | --- | --- | --- | --- | --- | --- |
| **Species** |  | **Mackerel** | |  | **Herring** | |  | **Blue whiting** | |  | **Mackerel** | |  | **Herring** | |  | **Blue whiting** | |
| **Season** |  | **May** | **July** |  | **May** | **July** |  | **May** | **July** |  | **May** | **July** |  | **May** | **July** |  | **May** | **July** |
| **N_stations_** |  | 0 | 14 |  | 23 | 0 |  | 24 | 7 |  | 0 | 0 |  | 7 | 5 |  | 7 | 0 |
| **N_fish_** |  | 0 | 115 |  | 191 | 0 |  | 190 | 54 |  | 0 | 0 |  | 61 | 20 |  | 63 | 0 |
| *Calanus finmarchicus* |  | - | 66.74 |  | 11.15 | - |  | 0.79 | 0 |  | - | - |  | 12.28 | 0.08 |  | 1.96E-05 | - |
| *Calanus glacialis* |  | - | 0 |  | 9.71E-03 | - |  | 0 | 0 |  | - | - |  | 0 | 0 |  | 0 | - |
| *Calanus hyperboreus* |  | - | 0 |  | 1.33 | - |  | 28.75 | 0 |  | - | - |  | 11.18 | 0 |  | 31.87 | - |
| *Calanus* spp. |  | - | 0 |  | 18.29 | - |  | 2.24 | 0 |  | - | - |  | 16.85 | 2.88 |  | 0.36 | - |
| *Clausocalanus* spp. |  | - | 0 |  | 1.39E-03 | - |  | 0 | 0 |  | - | - |  | 0 | 0 |  | 0 | - |
| *Euchaeta* spp. |  | - | 0 |  | 0 | - |  | 0.02 | 1.35 |  | - | - |  | 0 | 0 |  | 0.12 | - |
| *Metridia longa* |  | - | 0 |  | 0.22 | - |  | 1.15 | 0 |  | - | - |  | 6.00E-07 | 1.14E-05 |  | 0 | - |
| *Metridia* spp. |  | - | 0 |  | 0 | - |  | 0 | 0 |  | - | - |  | 0.47 | 0 |  | 6.53E-06 | - |
| *Calanoida ord. |  | - | 0 |  | 9.31 | - |  | 0.15 | 0.20 |  | - | - |  | 0.59 | 28.22 |  | 0 | - |
| *Copepoda subcl. |  | - | 0 |  | 0.27 | - |  | 0 | 0 |  | - | - |  | 0.56 | 0 |  | 6.22 | - |
| **Total copepods** |  | - | ***66.74*** |  | ***40.58*** | - |  | ***33.09*** | ***1.55*** |  | - | - |  | ***41.94*** | ***31.17*** |  | ***38.58*** | - |
| *Meganyctiphanes norvegica* |  | - | 0 |  | 0.11 | - |  | 9.15 | 41.19 |  | - | - |  | 0 | 0 |  | 0.44 | - |
| *Thysanoessa inermis* |  | - | 0 |  | 0 | - |  | 0 | 0 |  | - | - |  | 0 | 0 |  | 0 | - |
| *Thysanoessa longicaudata* |  | - | 0 |  | 0 | - |  | 0 | 8.12 |  | - | - |  | 0 | 0 |  | 0 | - |
| *Thysanoessa* spp. |  | - | 0 |  | 0 | - |  | 0 | 0 |  | - | - |  | 0 | 0 |  | 0 | - |
| *Euphausiacea ord. |  | - | 0.38 |  | 7.06 | - |  | 45.89 | 38.96 |  | - | - |  | 6.30 | 5.98 |  | 45.85 | - |
| **Total euphausiids** |  | - | ***0.38*** |  | ***7.17*** | - |  | ***55.04*** | ***88.27*** |  | - | - |  | ***6.30*** | ***5.98*** |  | ***46.29*** | - |
| *Pandalus* spp. |  | - | 0 |  | 0 | - |  | 0 | 0 |  | - | - |  | 4.47E-06 | 0 |  | 0 | - |
| *Caridea infraord. |  | - | 0 |  | 0 | - |  | 0 | 0 |  | - | - |  | 0.42 | 0.01 |  | 0 | - |
| *Decapoda ord. |  | - | 0 |  | 0 | - |  | 0 | 0 |  | - | - |  | 0 |  |  | 0 | - |
| **Total decapods** |  | - | ***0.00*** |  | ***0.00*** | - |  | ***0.00*** | ***0.00*** |  | - | - |  | ***0.42*** | ***0.01*** |  | ***0.00*** | - |
| *Themisto abyssorum* |  | - | 1.64 |  | 0.24 | - |  | 1.58 | 3.55 |  | - | - |  | 0.08 | 0.05 |  | 1.23 | - |
| *Themisto libellula* |  | - | 0 |  | 0.03 | - |  | 0 | 0 |  | - | - |  | 0 | 0 |  | 0 | - |
| *Themisto* spp. |  | - | 0 |  | 0 | - |  | 0 | 0 |  | - | - |  | 2.95 | 2.48 |  | 0.78 | - |
| *Hyperiidae fam. |  | - | 0 |  | 0 | - |  | 0 | 1.61 |  | - | - |  | 0 | 0 |  | 0 | - |
| *Amphipoda ord. |  | - | 0 |  | 3.49 | - |  | 5.32 | 3.07 |  | - | - |  | 0.03 | 0 |  | 1.10 | - |
| **Total amphipods** |  | - | ***1.64*** |  | ***3.76*** | - |  | ***6.90*** | ***8.22*** |  | - | - |  | ***3.07*** | ***2.52*** |  | ***3.11*** | - |
| *Evadne* spp. |  | - | 0 |  | 0 | - |  | 0 | 0 |  | - | - |  | 0 | 7.25E-05 |  | 0 | - |
| *Podon* spp. |  | - | 0 |  | 0 | - |  | 0 | 0 |  | - | - |  | 0 | 0 |  | 0 | - |
| *Cladocera infraord. |  | - | 0 |  | 0 | - |  | 0 | 0 |  | - | - |  | 0 | 0 |  | 0 | - |
| **Total cladocerans** |  | - | ***0.00*** |  | ***0.00*** | - |  | ***0.00*** | ***0.00*** |  | - | - |  | ***0.00*** | ***0.00*** |  | ***0.00*** | - |
| Ostracoda cl. |  | - | 0 |  | 0 | - |  | 0 | 0 |  | - | - |  | 0 | 0 |  | 0 | - |
| *Crustacea subph. |  | - | 0 |  | 0.54 | - |  | 0 | 0 |  | - | - |  | 0.34 | 0 |  | 0 | - |
|  |  |  |  |  |  |  |  |  |  |  |  |  |  |  |  |  |  |  |
| **Total crustaceans** |  | - | ***68.76*** |  | ***52.05*** | - |  | ***95.02*** | ***98.05*** |  | - | - |  | ***52.06*** | ***39.68*** |  | ***87.98*** | - |
|  |  |  |  |  |  |  |  |  |  |  |  |  |  |  |  |  |  |  |
| *Gonatus fabricii* |  | - | 0 |  | 0 | - |  | 0 | 0 |  | - | - |  | 0 | 0 |  | 0 | - |
| *Cephalopoda cl. |  | - | 0.17 |  | 0 | - |  | 0 | 0 |  | - | - |  | 0 | 0 |  | 0 | - |
| **Total cephalopods** |  | - | ***0.17*** |  | ***0.00*** | - |  | ***0.00*** | ***0.00*** |  | - | - |  | ***0.00*** | ***0.00*** |  | ***0.00*** | - |
| *Limacina retroversa* |  | - | 0.61 |  | 0.07 | - |  | 0 | 0 |  | - | - |  | 0 | 0 |  | 0 | - |
| *Limacina* spp. |  | - | 0 |  | 0 | - |  | 0 | 0 |  | - | - |  | 0 | 0 |  | 0 | - |
| *Gastropoda cl. |  | - | 0 |  | 0 | - |  | 0 | 0 |  | - | - |  | 0 | 0 |  | 0 | - |
| **Total gastropods** |  | ***-*** | ***0.61*** |  | ***0.07*** | ***-*** |  | ***0.00*** | ***0.00*** |  | ***-*** | ***-*** |  | ***0.00*** | ***0.00*** |  | ***0.00*** | ***-*** |
| Chaetognatha ph. |  | - | 0 |  | 6.80E-03 | - |  | 0 | 0 |  | - | - |  | 0 | 0 |  | 0 | - |
| Hydrozoa cl. |  | - | 0 |  | 0 | - |  | 0 | 0 |  | - | - |  | 0 | 0 |  | 0 | - |
|  |  |  |  |  |  |  |  |  |  |  |  |  |  |  |  |  |  |  |
| **Total invertebrates** |  | ***-*** | ***69.55*** |  | ***52.12*** | ***-*** |  | ***95.02*** | ***98.05*** |  | ***-*** | ***-*** |  | ***52.06*** | ***39.68*** |  | ***87.98*** | ***-*** |
|  |  |  |  |  |  |  |  |  |  |  |  |  |  |  |  |  |  |  |
| *Oikopleura* spp. |  | - | 0 |  | 0 | - |  | 0 | 0 |  | - | - |  | 0 | 60.32 |  | 0 | - |
| *Appendicularia cl. |  | - | 30.36 |  | 47.32 | - |  | 0 | 1.34 |  | - | - |  | 47.79 | 0 |  | 0.15 | - |
| **Total tunicates** |  | ***-*** | ***30.36*** |  | ***47.32*** | ***-*** |  | ***0.00*** | ***1.34*** |  | ***-*** | ***-*** |  | ***47.79*** | ***60.32*** |  | ***0.15*** | ***-*** |
| Myctophidae fam. |  | - | 0 |  | 0 | - |  | 2.12 | 0 |  | - | - |  | 0 | 0 |  | 11.44 | - |
| *Actinopterygii cl. |  | - | 0 |  | 0 | - |  | 1.00 | 0 |  | - | - |  | 0.08 | 0 |  | 0 | - |
| **Total teleostei** |  | ***-*** | ***0.00*** |  | ***0.00*** | ***-*** |  | ***3.12*** | ***0.00*** |  | ***-*** | ***-*** |  | ***0.08*** | ***0.00*** |  | ***11.44*** | ***-*** |
|  |  |  |  |  |  |  |  |  |  |  |  |  |  |  |  |  |  |  |
| **Total vertebrates** |  | ***-*** | ***30.36*** |  | ***47.32*** | ***-*** |  | ***3.12*** | ***1.34*** |  | ***-*** | ***-*** |  | ***47.87*** | ***60.32*** |  | ***11.59*** | ***-*** |
|  |  |  |  |  |  |  |  |  |  |  |  |  |  |  |  |  |  |  |
| Other remains |  | - | 0 |  | 0 | - |  | 0 | 0 |  | - | - |  | 0 | 0 |  | 0 | - |
| Unidentified remains |  | - | 0.09 |  | 0.56 | - |  | 1.86 | 0.62 |  | - | - |  | 0.07 | 0 |  | 0.43 | - |
|  |  |  |  |  |  |  |  |  |  |  |  |  |  |  |  |  |  |  |
| **Total** |  | ***-*** | ***100.00*** |  | ***100.00*** | ***-*** |  | ***100.00*** | ***100.00*** |  | ***-*** | ***-*** |  | ***100.00*** | ***100.00*** |  | ***100.00*** | ***-*** |

| **Year** |  | **2009** | | | | | | | |  | **2010** | | | | | | | |
| --- | --- | --- | --- | --- | --- | --- | --- | --- | --- | --- | --- | --- | --- | --- | --- | --- | --- | --- |
| **Species** |  | **Mackerel** | |  | **Herring** | |  | **Blue whiting** | |  | **Mackerel** | |  | **Herring** | |  | **Blue whiting** | |
| **Season** |  | **May** | **July** |  | **May** | **July** |  | **May** | **July** |  | **May** | **July** |  | **May** | **July** |  | **May** | **July** |
| **N_stations_** |  | 0 | 8 |  | 5 | 6 |  | 4 | 0 |  | 0 | 29 |  | 2 | 23 |  | 1 | 6 |
| **N_fish_** |  | 0 | 35 |  | 45 | 19 |  | 36 | 0 |  | 0 | 263 |  | 13 | 210 |  | 9 | 53 |
| *Calanus finmarchicus* |  | - | 22.90 |  | 1.54 | 8.21E-05 |  | 0 | - |  | - | 42.83 |  | 35.90 | 0.95 |  | 0 | 14.12 |
| *Calanus glacialis* |  | - | 0 |  | 4.73E-06 | 0 |  | 7.28E-03 | - |  | - | 0 |  | 0 | 0 |  | 0 | 0 |
| *Calanus hyperboreus* |  | - | 0 |  | 1.00 | 0 |  | 0.21 | - |  | - | 0 |  | 13.98 | 0.86 |  | 4.15 | 0.04 |
| *Calanus* spp. |  | - | 0.11 |  | 49.31 | 24.19 |  | 0.02 | - |  | - | 4.13 |  | 31.01 | 15.65 |  | 0 | 3.77E-06 |
| *Clausocalanus* spp. |  | - | 0 |  | 0 | 0 |  | 0 | - |  | - | 1.86E-09 |  | 0 | 8.11E-03 |  | 0 | 0 |
| *Euchaeta* spp. |  | - | 0 |  | 0 | 0 |  | 0.03 | - |  | - | 4.42E-03 |  | 0 | 0.22 |  | 0 | 17.03 |
| *Metridia longa* |  | - | 0 |  | 2.85 | 7.13E-06 |  | 0 | - |  | - | 0 |  | 0.41 | 0.49 |  | 0 | 0.02 |
| *Metridia* spp. |  | - | 0 |  | 1.73 | 0 |  | 0 | - |  | - | 0 |  | 0.62 | 0.50 |  | 0 | 0 |
| *Calanoida ord. |  | - | 3.20 |  | 4.86 | 23.50 |  | 0 | - |  | - | 0.33 |  | 0 | 34.24 |  | 0 | 0.46 |
| *Copepoda subcl. |  | - | 6.91E-06 |  | 0 | 0 |  | 0 | - |  | - | 2.35 |  | 0 | 8.13 |  | 0 | 0.02 |
| **Total copepods** |  | - | ***26.21*** |  | ***61.30*** | ***47.69*** |  | ***0.26*** | - |  | - | ***49.64*** |  | ***81.93*** | ***61.06*** |  | ***4.15*** | ***31.70*** |
| *Meganyctiphanes norvegica* |  | - | 0 |  | 0 | 0 |  | 10.14 | - |  | - | 0 |  | 0 | 0 |  | 2.33 | 0.22 |
| *Thysanoessa inermis* |  | - | 0 |  | 0 | 0 |  | 0 | - |  | - | 0 |  | 0 | 7.04E-07 |  | 5.88 | 0.38 |
| *Thysanoessa longicaudata* |  | - | 1.17E-05 |  | 0 | 0 |  | 0 | - |  | - | 0 |  | 0.93 | 2.03E-03 |  | 1.04 | 1.13 |
| *Thysanoessa* spp. |  | - | 0 |  | 0 | 0 |  | 0 | - |  | - | 0 |  | 0 | 1.62E-06 |  | 0 | 1.22 |
| *Euphausiacea ord. |  | - | 11.56 |  | 3.65 | 30.36 |  | 83.17 | - |  | - | 11.03 |  | 0.62 | 7.60 |  | 57.51 | 35.32 |
| **Total euphausiids** |  | - | ***11.56*** |  | ***3.65*** | ***30.36*** |  | ***93.31*** | - |  | - | ***11.03*** |  | ***1.55*** | ***7.60*** |  | ***66.75*** | ***38.28*** |
| *Pandalus* spp. |  | - | 0 |  | 0 | 0 |  | 0 | - |  | - | 0 |  | 0 | 0 |  | 0 | 0 |
| *Caridea infraord. |  | - | 0 |  | 0 | 0 |  | 0 | - |  | - | 2.23E-03 |  | 0 | 0 |  | 0 | 0 |
| *Decapoda ord. |  | - | 0 |  | 0 | 0 |  | 0 | - |  | - | 0 |  | 0 | 0.56 |  | 0 | 0 |
| **Total decapods** |  | - | ***0.00*** |  | ***0.00*** | ***0.00*** |  | ***0.00*** | - |  | - | ***0.00*** |  | ***0.00*** | ***0.56*** |  | ***0.00*** | ***0.00*** |
| *Themisto abyssorum* |  | - | 1.03 |  | 0.14 | 17.52 |  | 0.62 | - |  | - | 0.90 |  | 1.45 | 4.58 |  | 2.80 | 9.00 |
| *Themisto libellula* |  | - | 0 |  | 0 | 8.91E-03 |  | 0 | - |  | - | 4.65E-03 |  | 0.62 | 0.88 |  | 0.09 | 1.38 |
| *Themisto* spp. |  | - | 2.09E-08 |  | 0 | 4.31 |  | 0 | - |  | - | 0.23 |  | 1.52 | 7.03 |  | 23.51 | 18.56 |
| *Hyperiidae fam. |  | - | 1.34E-06 |  | 0 | 0 |  | 0 | - |  | - | 0 |  | 0 | 0 |  | 0 | 0 |
| *Amphipoda ord. |  | - | 0.03 |  | 0.12 | 0 |  | 5.81 | - |  | - | 0 |  | 0 | 0 |  | 0 | 0 |
| **Total amphipods** |  | - | ***1.06*** |  | ***0.26*** | ***21.84*** |  | ***6.43*** | - |  | - | ***1.14*** |  | ***3.59*** | ***12.49*** |  | ***26.40*** | ***28.94*** |
| *Evadne* spp. |  | - | 50.15 |  | 0 | 1.47E-05 |  | 0 | - |  | - | 0.07 |  | 0 | 1.60 |  | 0 | 0 |
| *Podon* spp. |  | - | 0 |  | 0 | 0 |  | 0 | - |  | - | 1.86E-10 |  | 0 | 2.03E-07 |  | 0 | 0 |
| *Cladocera infraord. |  | - | 5.51E-04 |  | 0 | 0 |  | 0 | - |  | - | 8.92E-03 |  | 0 | 1.01E-07 |  | 0 | 0 |
| **Total cladocerans** |  | - | ***50.15*** |  | ***0.00*** | ***0.00*** |  | ***0.00*** | - |  | - | ***0.08*** |  | ***0.00*** | ***1.60*** |  | ***0.00*** | ***0.00*** |
| Ostracoda cl. |  | - | 0 |  | 0 | 0 |  | 0 | - |  | - | 0 |  | 0 | 0.04 |  | 0 | 0 |
| *Crustacea subph. |  | - | 0 |  | 0 | 0 |  | 0 | - |  | - | 2.25E-03 |  | 0 | 1.52 |  | 0 | 1.08 |
|  |  |  |  |  |  |  |  |  |  |  |  |  |  |  |  |  |  |  |
| **Total crustaceans** |  | - | ***88.98*** |  | ***65.21*** | ***99.89*** |  | ***100.00*** | - |  | - | ***61.89*** |  | ***87.07*** | ***84.87*** |  | ***97.30*** | ***100.00*** |
|  |  |  |  |  |  |  |  |  |  |  |  |  |  |  |  |  |  |  |
| *Gonatus fabricii* |  | - | 0 |  | 0 | 0 |  | 0 | - |  | - | 0 |  | 0 | 0 |  | 0 | 0 |
| *Cephalopoda cl. |  | - | 0.87 |  | 0 | 0 |  | 0 | - |  | - | 0.34 |  | 12.93 | 0 |  | 1.16 | 0 |
| **Total cephalopods** |  | - | ***0.87*** |  | ***0.00*** | ***0.00*** |  | ***0.00*** | - |  | - | ***0.34*** |  | ***12.93*** | ***0.00*** |  | ***1.16*** | ***0.00*** |
| *Limacina retroversa* |  | - | 3.40E-05 |  | 6.70E-06 | 1.58E-07 |  | 0 | - |  | - | 5.88 |  | 0 | 0.06 |  | 0 | 0 |
| *Limacina* spp. |  | - | 0 |  | 0 | 0 |  | 0 | - |  | - | 2.79E-04 |  | 0 | 4.06E-03 |  | 0 | 0 |
| *Gastropoda cl. |  | - | 1.41E-07 |  | 0 | 0 |  | 0 | - |  | - | 1.07E-03 |  | 0 | 0 |  | 0 | 0 |
| **Total gastropods** |  | ***-*** | ***0.00*** |  | ***0.00*** | ***0.00*** |  | ***0.00*** | ***-*** |  | ***-*** | ***5.88*** |  | ***0.00*** | ***0.07*** |  | ***0.00*** | ***0.00*** |
| Chaetognatha ph. |  | - | 0 |  | 0 | 0.09 |  | 0 | - |  | - | 0 |  | 0 | 0.25 |  | 0 | 0 |
| Hydrozoa cl. |  | - | 0 |  | 0 | 0 |  | 0 | - |  | - | 0 |  | 0 | 0 |  | 0 | 0 |
|  |  |  |  |  |  |  |  |  |  |  |  |  |  |  |  |  |  |  |
| **Total invertebrates** |  | ***-*** | ***89.85*** |  | ***65.21*** | ***99.98*** |  | ***100.00*** | ***-*** |  | ***-*** | ***68.11*** |  | ***100.00*** | ***85.19*** |  | ***98.46*** | ***100.00*** |
|  |  |  |  |  |  |  |  |  |  |  |  |  |  |  |  |  |  |  |
| *Oikopleura* spp. |  | - | 4.62 |  | 0 | 0.02 |  | 0 | - |  | - | 3.11 |  | 0 | 2.62 |  | 0 | 0 |
| *Appendicularia cl. |  | - | 4.58 |  | 31.94 | 0 |  | 0 | - |  | - | 27.81 |  | 0 | 11.88 |  | 0 | 0 |
| **Total tunicates** |  | ***-*** | ***9.20*** |  | ***31.94*** | ***0.02*** |  | ***0.00*** | ***-*** |  | ***-*** | ***30.92*** |  | ***0.00*** | ***14.51*** |  | ***0.00*** | ***0.00*** |
| Myctophidae fam. |  | - | 0 |  | 0 | 0 |  | 0 | - |  | - | 0 |  | 0 | 0 |  | 0 | 0 |
| *Actinopterygii cl. |  | - | 0 |  | 0 | 0 |  | 0 | - |  | - | 0.85 |  | 0 | 0 |  | 1.54 | 0 |
| **Total teleostei** |  | ***-*** | ***0.00*** |  | ***0.00*** | ***0.00*** |  | ***0.00*** | ***-*** |  | ***-*** | ***0.85*** |  | ***0.00*** | ***0.00*** |  | ***1.54*** | ***0.00*** |
|  |  |  |  |  |  |  |  |  |  |  |  |  |  |  |  |  |  |  |
| **Total vertebrates** |  | ***-*** | ***9.20*** |  | ***31.94*** | ***0.02*** |  | ***0.00*** | ***-*** |  | ***-*** | ***31.77*** |  | ***0.00*** | ***14.51*** |  | ***1.54*** | ***0.00*** |
|  |  |  |  |  |  |  |  |  |  |  |  |  |  |  |  |  |  |  |
| Other remains |  | - | 1.34E-06 |  | 0 | 0 |  | 0 | - |  | - | 6.20E-08 |  | 0 | 2.25E-03 |  | 0 |  |
| Unidentified remains |  | - | 0.95 |  | 2.86 | 1.74E-05 |  | 0 | - |  | - | 0.13 |  | 0 | 0.31 |  | 0 | 4.71E-06 |
|  |  |  |  |  |  |  |  |  |  |  |  |  |  |  |  |  |  |  |
| **Total** |  | ***-*** | ***100.00*** |  | ***100.00*** | ***100.00*** |  | ***100.00*** | ***-*** |  | ***-*** | ***100.00*** |  | ***100.00*** | ***100.00*** |  | ***100.00*** | ***100.00*** |
